# A small-molecule HSP90 inhibitor, NVP-HSP990, alleviates rotavirus infection

**DOI:** 10.1128/jvi.01883-25

**Published:** 2025-12-10

**Authors:** Yi Cao, Qingmin Zhu, Xiaoping Wu, Zhunyi Xie, Chengying Yang, Yanyan Guo, Dongwei Meng, Xinyuan Zhou, Yuzhang Wu, Jintao Li, Haiyang He

**Affiliations:** 1 Department of Immunology, Basic Medical School, Army Medical University (Third Military Medical University) 12525, Chongqing, China; 2 Team 13 of the Fourth Brigade, Basic Medical School, Army Medical University (Third Military Medical University) 12525, Chongqing, China; 3 Institute of Cancer, Xinqiao Hospital, Army Medical University (Third Military Medical University) 12525, Chongqing, China; 4 Institute for Cancer Medicine and School of Basic Medical Sciences, Southwest Medical University 74647, Luzhou, Sichuan, China; 5 Department of Biosafety, Basic Medical School, Army Medical University (Third Military Medical University) 12525, Chongqing, China; University of Michigan Medical School, Ann Arbor, Michigan, USA

**Keywords:** diarrhea, MAPK signaling pathway, NVP-HSP990, rotavirus, tight junctions

## Abstract

Rotavirus (RV) infection remains a leading cause of hospitalization and mortality among infants and young children. Despite global implementation of RV vaccines, hundreds of thousands of infants and young children still succumb to this disease each year due to ineffective treatment. In this study, we demonstrated that NVP-HSP990, a novel small-molecule heat shock protein 90 (HSP90) inhibitor, inhibited RV infection with a fascinatingly higher selectivity index compared to conventional HSP90 inhibitors like geldanamycin and its derivative tanespimycin (17-allylamino-17-demethoxygeldanamycin [17-AAG]). NVP-HSP990 effectively inhibited RV replication *in vitro* without blocking the initial establishment of infection. NVP-HSP990 restored host gene expression in most KEGG pathways disrupted by RV infection in Caco-2 cells, except some inflammatory pathways (such as IL-17 and TNF pathways). NVP-HSP990 significantly inhibited RV-induced activation of the MAPK pathway and prevented the disruption of tight junctions in Caco-2 cells. More importantly, NVP-HSP990 effectively suppressed RV infection in BALB/c suckling mice and significantly alleviated RV-induced diarrhea.

**IMPORTANCE:**

Rotavirus (RV) infection poses a global health threat with an urgent need for targeted antiviral therapies. Here, we identified NVP-HSP990 as a next-generation HSP90 inhibitor with exceptional translational potential against RV infection. Compared to conventional HSP90 inhibitors, NVP-HSP990 demonstrated markedly enhanced anti-RV selectivity. NVP-HSP990 effectively reversed dysregulation of key host pathways in RV infection while selectively modulating pro-inflammatory responses, thereby balancing antiviral and immunopathological outcomes. NVP-HSP990 also blocked MAPK-driven tight junction disruption to preserve intestinal barrier integrity. As a result, NVP-HSP990 significantly alleviated the severity of RV-induced diarrhea. Given its excellent oral efficacy and systemic penetration previously reported, NVP-HSP990 emerges as a promising HSP90-targeted candidate capable of addressing both intestinal and possible extraintestinal RV infections, which also repositions HSP90 inhibition as a viable strategy in RV management.

## INTRODUCTION

Rotavirus (RV), a double-stranded RNA virus that belongs to the Sedoreoviridae family (1), is the leading cause of diarrhea in infants and young children (2). RV infection mainly occurs in the intestinal epithelium and causes dysfunction of enterocytes and disorder of the enteric nervous system, leading to severe dehydrating diarrhea and vomiting; moreover, beyond gastrointestinal symptoms, severe RV infection sometimes causes systemic infection through viremia, affecting critical organs such as the brain and leading to poor prognosis (3–5). Although expansion of RV vaccines into national immunization programs worldwide has led to a 59% decrease in hospitalizations and a 36% decrease in deaths caused by RV infection (6), according to the latest global survey by WHO, RV infection still causes approximately 0.64% of hospitalizations and 208,009 deaths of children under 5 years of age in 28 low- and middle-income countries (7). Symptomatic treatments and gastrointestinal protective agents are usually the main choices for management of RV infection, while conventional antiviral drugs like nucleoside analogs and type I interferon are rarely employed due to their significant adverse side effects (8, 9). This limitation sometimes results in uncontrolled RV infections and even mortality. Therefore, there is an urgent need to develop safe and effective antiviral drugs for RV infections.

Heat shock protein 90 (HSP90), a chaperone protein present in both eukaryotes and bacteria, has four isoforms: HSP90α, HSP90β, GRP94, and TRAP1. HSP90α and HSP90β are predominantly found in the cytoplasm, GRP94 is located in the endoplasmic reticulum, and TRAP1 is present in the mitochondria (10, 11). HSP90 promotes viral replication by regulating cell signaling systems or by directly interacting with viral proteins such as hepatitis B virus reverse transcriptase, hepatitis C virus non-structural protein 5A, and influenza virus A RNA-dependent RNA polymerase (12–15). HSP90 is involved in the entry of RV into certain tumor cell lines (16–18), and also contributes to RV replication *in vitro* (19, 20). Therefore, it seems that HSP90 inhibitors would be promising broad-spectrum antiviral drug candidates (21); nevertheless, in contrast to their widespread application in anti-tumor therapies (22), no HSP90 inhibitors are in clinical use for antiviral therapies currently, probably due to unsatisfactory antiviral efficacy and undesirable toxicity.

NVP-HSP990 is a novel small-molecule HSP90 inhibitor fundamentally distinct from the natural HSP90 inhibitor geldanamycin (GA) and its derivative tanespimycin (17-allylamino-17-demethoxygeldanamycin, 17-AAG) in both chemical structure and mechanism (23–25). Briefly, as a fully synthetic inhibitor, NVP-HSP990 adopts a resorcinol-isoxazole scaffold, eliminating the natural-product-derived macrocyclic benzoquinone ansamycin backbone shared by GA and 17-AAG. This synthetic design avoids the redox-active benzoquinone moiety responsible for oxidative stress and hepatotoxicity in ansamycins. While GA and 17-AAG rely on a 19-membered macrocycle and benzoquinone for ATPase binding, NVP-HSP990 achieves potent HSP90 inhibition through its optimized resorcinol-based architecture. Critically, NVP-HSP990 exhibits superior oral bioavailability without quinone-driven toxicity, distinguishing it from intravenous-administered ansamycins and positioning it as a next-generation HSP90 inhibitor with improved safety and therapeutic potential (26). These advantages position NVP-HSP990 as a promising antiviral candidate even though experimental data on its effects against viral infections are currently limited.

In this study, we aimed to investigate the inhibitory effects of NVP-HSP990 on RV infection. Specifically, we evaluated its antiviral efficacy against RV *in vitro* and *in vivo*, elucidated its potential mechanisms of action, and assessed its therapeutic efficacy on RV diarrhea, thereby providing a solid foundation for the development of NVP-HSP990 as a promising therapeutic agent against RV infection.

## MATERIALS AND METHODS

### Cell culture, viral infection, and drug administration

Rhesus monkey embryo kidney cell line MA104 cells (ATCC: CRL-2378.1) were provided by Dr. Elschner (Friedrich-Loeffler-Institute). Human intestinal epithelial cell lines Caco-2 cells (ATCC: HTB-37) and HT-29 cells (ATCC: HTB-38) were from ATCC and kept in our institute. All the cells were cultured in complete DMEM (Dulbecco’s modified Eagle medium [Gibco, USA] plus 10% [vol/vol] fetal bovine serum [FBS] [Gibco, USA] and 1% penicillin/streptomycin [Gibco, USA]) at 37°C with 5% CO_2_. RV Wa (G1P[8]) and SA11 (G3P[2]) strains were gifted by Professor Duan Zhaojun from the China Center for Disease Control and Prevention (Beijing, China) and propagated in MA104 cells. RV EDIM strain G16P[16] was gifted by Professor Wan Jianwei in Christophe Merieux Laboratory (Beijing) and propagated in BALB/c suckling mice. For RV infection *in vitro*, the viruses were diluted in DMEM, activated with 10 µg/mL trypsin (Gibco, USA) for 30 min at 37°C, and then added to target cells previously washed once with DMEM. After 1 h of incubation at 37°C, the inoculum was removed; the cells were then washed twice with DMEM (without FBS) and incubated with DMEM (without FBS) at 37°C, which was denoted as 0 h post-infection (h p.i.). HSP90 inhibitors (NVP-HSP990, GA, and 17-AAG) and Ribavirin (Selleck, USA) were dissolved in DMSO (Sigma, USA). The drugs were administered with indicated concentrations at 0 h p.i. *in vitro* unless otherwise stated, or orally administered in 10 µL volume using a pipette tip with the indicated quantity plus 90% (vol/vol) corn oil.

### Cytotoxicity assay

To evaluate the cytotoxicity of different HSP90 inhibitors *in vitro*, 10,000 MA104 cells, 30,000 Caco-2 cells, and 80,000 HT-29 cells in 100 µL complete DMEM medium were seeded in 96-well plates and cultured at 37°C and 5% CO_2_ for 24 h to reach about 100% confluence, and a cell-free control was also set. After removing the initial medium, 100 µL of complete DMEM medium containing the indicated concentrations of drugs was added to each well, and the plates were incubated at 37°C and 5% CO_2_. Twenty-four hours later, the drug-containing medium was replaced with 100 µL fresh complete DMEM medium plus 10 µL 5 mM CCK-8 reagent (Beyotime, China), and OD_450_ absorbance of each well was detected 3 h later using a microplate reader (Gene Company Limited, China). Relative cell viability rate = OD_450_ (drug-treated cells − cell-free control) / OD_450_ (0 µM drug-treated cells − cell-free control).

### *In vitro* assay of viral inhibitory effects of drugs

For analysis of effects of different HSP990 inhibitors on RV replication *in vitro*, 10,000 MA104 cells, 30,000 Caco-2 cells, and 80,000 HT-29 cells in 100 µL complete DMEM medium were seeded in 96-well plates and cultured at 37°C and 5% CO_2_ for 24 h. Then the cells were infected with RV Wa or SA11 strains (MOI = 1 (PFU/cell)) for 1 h. After removing the viruses and washing cells two times with DMEM, 100 µL DMEM containing the indicated concentrations of drugs was added to each well, and the plates were incubated at 37°C and 5% CO_2_. At 24 h p.i., the infected cells and culture medium were collected, frozen/thawed twice, and subjected to centrifugation at 1,000 *× g* for 3 min at room temperature. Then, the supernatant was subjected to analysis of viral load by PFA. The 50% inhibitory concentration (IC_50_) values were determined by nonlinear regression (curve fit) analysis in GraphPad Prism 9.0 (GraphPad Software). Dose-response data (drug concentrations as *x*-axis and normalized inhibition percentages as *y*-axis) were fitted to a dose-response inhibition model ([Inhibitor] vs normalized response – Variable slope). The curve was constrained to a bottom limit of 0% inhibition. The IC_50_ value was automatically calculated as the *x*-intercept at *y* = 50%. Model adequacy was validated using goodness-of-fit metrics (*R*^2^ ≥ 0.95), 95% confidence intervals, and residual distribution analysis.

### RNA-sequencing (RNA-seq) analysis

Caco-2 cells in six-well tissue culture plates (90% confluence) were infected with RV Wa or SA11 strains (MOI = 3) for 1 h. After removing the viruses and washing cells two times with DMEM, the cells were cultivated with 2 mL DMEM containing 100 nM NVP-HSP990 or an equal volume of DMSO at 37°C and 5% CO_2_. At 24 h p.i., the Caco-2 cells were harvested, and total RNA was extracted using Trizol reagent kit (Invitrogen, USA), according to the manufacturer’s protocol. RNA quality was assessed on an Agilent 2100 Bioanalyzer (Agilent Technologies, Palo Alto, CA, USA) and checked using RNase-free agarose gel electrophoresis. After total RNA was extracted, eukaryotic mRNA was enriched by Oligo(dT) beads and used for library construction and next-generation sequencing.

### Immunofluorescence assay (IFA)

Caco-2 cells growing on coverslips were mock-infected with PBS or infected with RV Wa or SA11 strains (MOI = 1), then the cells were cultivated with DMEM containing 100 nM NVP-HSP990 or an equal volume of DMSO as a control for another 18 h after infection. The cells were then washed with PBS, fixed in 4% paraformaldehyde for 20 min, followed by permeabilization in 0.5% Triton X-100 for 10 min and blocking with 5% BSA (Beyotime, China) for 1 h at room temperature. The cells were then incubated with rabbit anti-ZO-1 monoclonal antibody (CST, USA) (1:100) or rabbit anti-RV VP6 polyclonal antibodies (CUSABIO, China) (1:50) for 2 h at room temperature, washed twice with PBS, and incubated with Cy3-conjugated goat anti-rabbit antibodies (Beyotime, China) (1:500) for 1 h at room temperature. In some experiments, the cells were also stained with FITC-conjugated goat anti-RV antibodies (Virostat, USA) (1:100) for 1 h at room temperature and followed by 5 µg/mL 2-(4-amidinophenyl)-6-indolecarbamidine dihydrochloride (DAPI) (Sigma-Aldrich, USA) staining for 10 min. The triple-stained cells were washed twice with PBS and mounted in Prolong Gold Antifade Reagent (CST, USA). Microscopic images were acquired with an EVOS M5000 system (Thermo Fisher Scientific, USA) using the built-in Celleste software (v.6.0). Raw image files were saved in TIFF format to preserve data integrity. Minimal post-processing (image cropping and annotations) was applied through Adobe Illustrator 2019 (Adobe Systems, USA) to ensure readability. No alterations were made to raw data during this process.

### Western blot

MA104, Caco-2, and HT-29 cells were mock-infected with PBS, infected with RV Wa or SA11 strains (MOI = 3), or treated with 1 µM C16-PAF (C16) for 1 h. After removing the viruses or C16-PAF and washing cells two times with DMEM, the cells were cultivated with 2 mL DMEM containing 100 nM NVP-HSP990 (+), an equal volume of DMSO (−), or 1 µM C16-PAF at 37°C and 5% CO_2_ for 20 h. Then, the cells in each well (~5 × 10^5^ cells) were lysed with 100 µL RIPA lysis buffer (Beyotime, China) plus protease inhibitor cocktail (Thermo Fisher Scientific, USA). The cell lysates were collected in 1.5 mL EP tubes, sonicated five times for 15 s at 80 watts on ice, and clarified by centrifugation at 12,000 *× g* for 10 min at 4°C. After protein quantification with a BCA kit (Beyotime, China), the protein samples were mixed with 25 µL 5× SDS loading buffer (Beyotime, China), boiled at 100°C for 5 min, chilled on ice, vortexed for seconds, and centrifuged at 12,000 *× g* for 3 min at room temperature. Twenty micrograms of protein was subjected to protein electrophoresis in precast 4–20% SDS-PAGE gels (Beyotime, China), and then transferred to 0.22 µm PVDF membranes (Millipore, USA). After blocking with 5% BSA, the membranes were incubated with rabbit antibodies to MAPK components (SAPK/JNK, phospho-SAPK/JNK, p38 MAPK, phospho-p38 MAPK, ERK1/2, phospho-ERK1/2) (CST, USA), rabbit anti-RV VP6/VP7 polyclonal antibodies (CUSABIO, China), or mouse mAb to β-actin (Servicebio, China) as an internal reference. Afterward, the membranes were incubated with horseradish peroxidase-conjugated rat anti-rabbit IgG (CST, USA) or rat anti-mouse IgG (CST, USA). Immunoreactive bands were visualized using enhanced chemiluminescence substrate BeyoECL Plus (Beyotime, China).

### RV diarrhea models in suckling mice

BALB/c mice were purchased from Gempharmatech Inc. (Suzhou, China) and housed under SPF conditions (22 ± 1°C, 12 h light/dark cycle). Suckling mice were obtained by mating male and female adult BALB/c mice (12- to 16-week-old). Only the 7-day-old BALB/c suckling mice with body weights ranging from 2.5 g to 4.5 g were selected for RV diarrhea models. For induction of RV diarrhea, 1 × 10^6^ PFU of SA11 strain or 10 × DD_50_ of EDIM strain in 20 µL PBS was orally inoculated using a pipette tip to 7-day-old BALB/c suckling mice regardless of their sexes, as sex has no significant influence on RV diarrhea occurrence. We used a scoring system to evaluate fecal consistency and color as described with some modification (27): no stool or brown formed stool (1 point); brown soft stool (2 points); yellow soft stool (3 points); and yellow watery stool (4 points). Mice with scores of ≥2 meant diarrhea occurrence, while mice with scores of 1 meant no diarrhea. At the end of each experiment, all infected or drug-treated suckling mice were humanely euthanized via CO_2_ asphyxiation.

### *In vivo* RV inhibition assays

Seven-day-old BALB/c suckling mice meeting the weight criteria within each litter were randomly assigned to experimental groups. Each litter was allocated to ensure one to three suckling mice per experimental group (variations dependent on the total number of groups). Data from suckling mice assigned to the same group across multiple litters were aggregated to achieve a total sample size of *n* = 3–6 per experimental group. Then, the suckling mice were orally inoculated with 1 × 10^6^ PFU of the SA11 strain or 10 × DD_50_ of the EDIM strain, or with an equal amount of PBS as mock infection. For SA11 infection, the suckling mice were orally treated once with indicated doses of drugs or with an equal volume of DMSO as control at 2 h p.i. For EDIM infection, the suckling mice were orally treated with 0.2 mg/kg/day NVP-HSP990, 30 mg/kg/day ribavirin, or an equal amount of DMSO as control from 2 days post-infection (d p.i.) to indicated time points. Diarrhea scores and body weight were monitored from day 0 to day 7 post-infection in the SA11 infection model, and from day 0 to day 9 post-diarrhea occurrence (2–11 d p.i.) in the EDIM infection model. Blinding was applied throughout data collection and analysis.

The RV-infected suckling mice were sacrificed by CO_2_ asphyxiation at the indicated times, and small intestines (including duodenum, jejunum, and ileum) or colon was homogenized in 0.3 mL DMEM, centrifuged at 12,000 *× g* for 5 min at 4°C, and the supernatant was collected. Virus contents in the supernatant were titrated with PFA as described above. Viral antigens in the supernatant were detected with enzyme-linked immunosorbent assay (ELISA) kits for RV (CUSABIO, China) following the manufacturer’s instructions. For qPCR analysis of RV protein expression, the intestines were longitudinally split and washed in 1 mL DMEM by shaking for 30 s. Then, the tissues were cut into 0.5 cm segments, incubated in Hank’s buffer containing 2.5 mM EDTA-Na_2_ and 1 mM dithiothreitol (Sangon Biotech, China) at 37°C with rotation at 200 rpm for 30 min, filtered with a 70 µm strainer. Then the epithelial cells were pelleted by centrifugation at 500 *× g* for 5 min, purified with 20% Percoll (Cytiva, Sweden), and lysed in Trizol for RNA extraction and further qPCR analysis.

### Histopathology and immunohistochemistry

The jejunum and ileum of suckling mice were fixed in 4% paraformaldehyde for 24 h, washed with PBS twice, and then subjected to paraffin embedding and slicing at 5 µm thickness. The sections were stained with hematoxylin and eosin (Beyotime, China) and observed with an Olympus BX51 microscope, and the data were processed by CaseViewer 2.4 software. For immunohistochemistry analysis, the fixed tissues were dehydrated in 30% sucrose, embedded in Optimal Cutting Temperature compound, frozen with liquid nitrogen, and cryosliced at 10 µm thickness. The slices were air-dried, followed by antigen retrieval using 0.01 M citrate buffer (pH 6.0) at 100°C for 30 min, permeabilization in 0.5% Triton X-100 for 10 min, and blocking with 5% BSA (Beyotime, China) for 1 h at room temperature. Then, the slices were incubated with rabbit anti-RV VP7 polyclonal antibodies (CUSABIO, China) (1:200) overnight at 4°C, washed twice with PBS, incubated with FITC-conjugated goat anti-rabbit antibodies (ebioscience, USA) (1:500) for 1 h at room temperature and followed by 5 µg/mL DAPI (Sigma-Aldrich, USA) staining for 10 min. The double-stained slices were washed twice with PBS and mounted in Prolong Gold Antifade Reagent (CST, USA). Microscopic images were acquired with an EVOS M5000 system (Thermo Fisher Scientific, USA) using the built-in Celleste software (v.6.0). All raw image files were saved in TIFF format to preserve data integrity. Minimal post-processing (image cropping and annotations) was applied through Adobe Illustrator 2019 (Adobe Systems, USA) to ensure readability. No alterations were made to raw data during this process.

### Statistical analysis

Data were presented as means ± SEM. Statistical analysis was performed with Prism 9.0 (GraphPad). Paired Student’s *t*-test was used for comparisons between two matched groups. One-way analysis of variance (ANOVA) was used for comparisons between multiple groups. Two-way ANOVA was used for comparisons of grouped data. *P*-values <0.05 were considered significant (*P < 0.05; **P < 0.01; ***P < 0.001; ****P < 0.0001); ns, not significant, *P* > 0.05.

## RESULTS

### Antiviral activity of NVP-HSP990 *in vitro*

**Fig 1:**
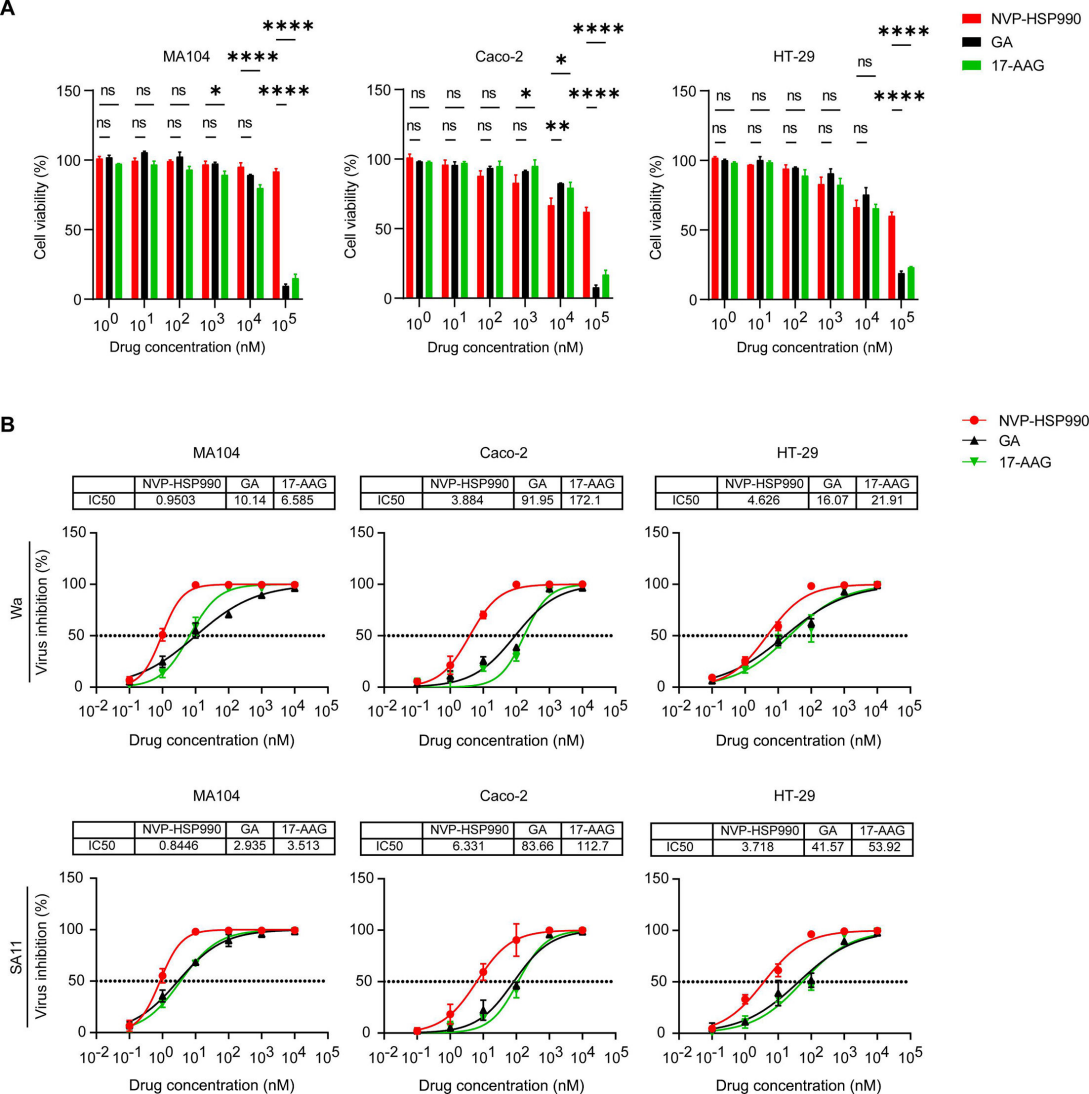
Cytotoxicity and inhibitory effects on RV replication of different HSP90 inhibitors. (**A**) Plots for cell viability of MA104, Caco-2, and HT-29 cells after treatment with NVP-HSP990, GA, or 17-AAG at indicated concentrations for 24 h. Cell viability was tested using the CCK-8 assay. (**B**) Plots for RV (Wa and SA11 strains) inhibition in MA104, Caco-2, and HT-29 cells after treatment with NVP-HSP990, GA, or 17-AAG at indicated concentrations for 24 h. RV replication was tested by PFA, and IC_50_ values are indicated at the top of each plot. The experiments were performed in triplicate, and the data are presented as mean ± SEM and are representative of four (**A**) and two (**B**) independent experiments. ns, not significant; *P < 0.05, **P < 0.01, ****P < 0.0001 (two-way ANOVA).

NVP-HSP990 is much smaller than and structurally different from the traditional HSP90 inhibitors GA and 17-AAG (Fig. S1). NVP-HSP990 showed much less cytotoxicity than GA and 17-AAG at 100 µM, with a CC_50_ (concentration of cytotoxicity 50%) >100 µM on all the cells; in contrast, the CC_50_ values of both GA and 17-AAG were <40 µM (Fig. 1A; Fig. S2, Table S2). NVP-HSP990 inhibited potent RV inhibition with IC_50_ (half-maximal inhibitory concentration) values of 0.96 ± 0.13 nM (Wa strain) and 0.84 ± 0.17 nM (SA11 strain) in MA104 cells, 4.00 ± 1.28 nM (Wa strain) and 7.47 ± 4.84 nM (SA11 strain) in Caco-2 cells, and 4.69 ± 0.90 nM (Wa strain) and 3.85 ± 1.16 nM (SA11 strain) in HT-29 cells, which were all significantly smaller than those of GA and 17-AAG (Fig. 1B; Table S2). Therefore, the selectivity index (SI) of NVP-HSP990 in RV infection was much higher than that of GA or 17-AAG in all tested cell lines (Table S2).

**Fig 2:**
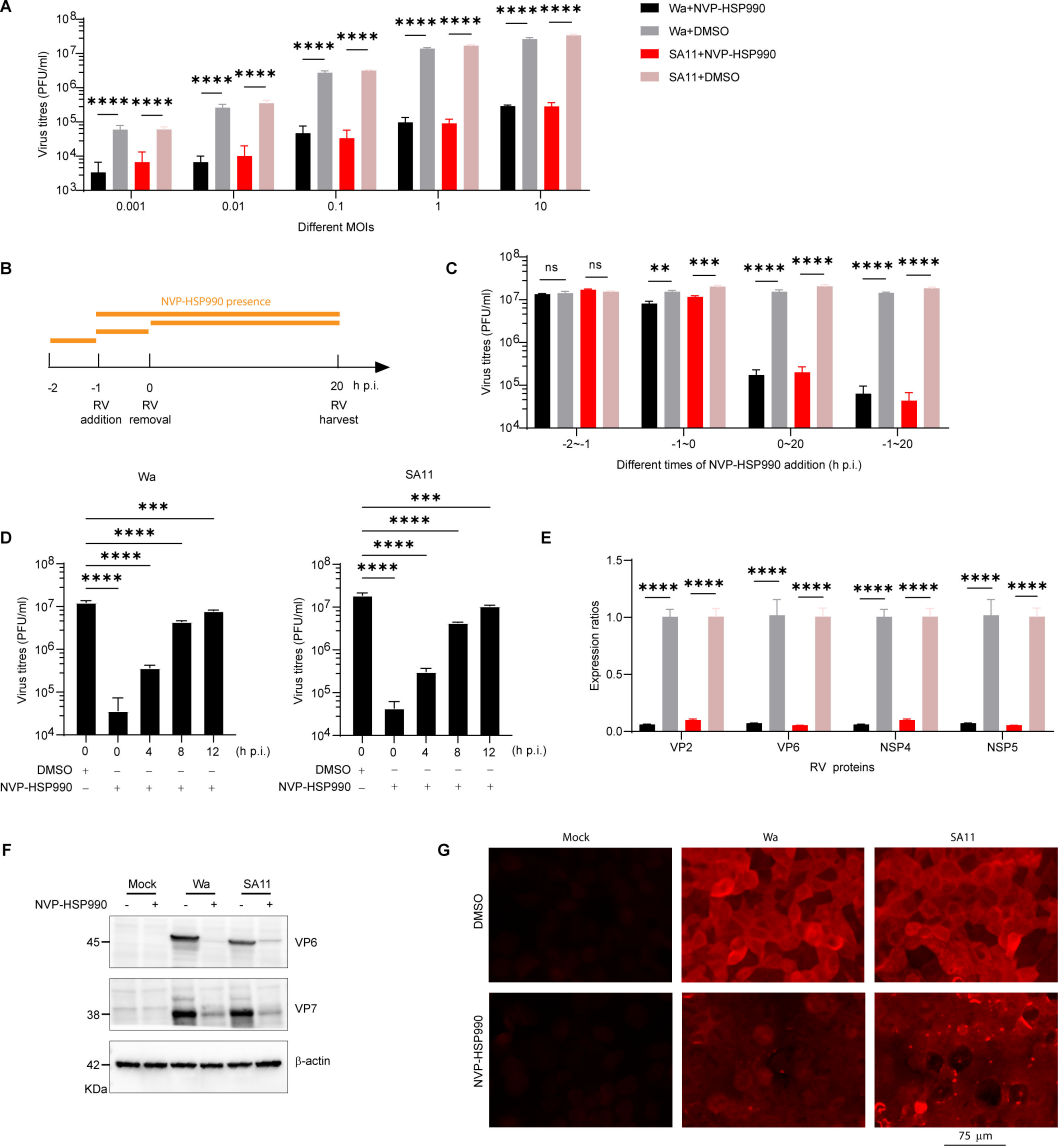
NVP-HSP990 significantly inhibited RV replication, viral gene transcription, and antigen expression. (**A**) Caco-2 cells were infected with RV (Wa or SA11 strains) at varying MOIs (0.001–10), followed by treatment with 100 nM NVP-HSP990 or an equal volume of DMSO (as a control) for 24 h. Viral replication was assessed using PFA. (**B**) Schematic diagram illustrating the timing of NVP-HSP990 addition and removal. (**C**) Caco-2 cells were infected with RV (MOI = 1) and treated with 100 nM NVP-HSP990 or with DMSO as control at indicated infection periods as shown in panel B. Viral replication was tested with PFA at 20 h p.i. (**D**) Caco-2 cells were infected with RV (MOI = 1) and treated with 100 nM HSP990 at 0, 4, 8, 12 h p.i. or treated with DMSO at 0 h p.i. Virus replication was tested with PFA at 24 h p.i. (**E**) Caco-2 cells were infected with RV (MOI = 1) and further cultivated with DMEM containing 100 nM NVP-HSP990 or DMSO as control for 18 h. Then the infected cells were harvested for qPCR analysis of the expression of RV genes encoding the VP2, VP6, NSP4, and NSP5 proteins. (**F**) Caco-2 cells were mock-infected with PBS or infected with RV (MOI = 1) and further cultivated with DMEM containing 100 nM NVP-HSP990 or DMSO as control for 18 h. Then the cells were harvested for WB analysis of RV structural proteins VP6 and VP7. (**G**) Caco-2 cells growing on coverslips were mock-infected with PBS or infected with RV (MOI = 3) and treated with 100 nM NVP-HSP990 or DMSO as control for 18 h. Then the infected cells were subjected to immunostaining of RV VP6 antigens (red). Data are presented as mean ± SEM (**A, C, D, E**). The experiments were performed in triplicate (**A, C, D, E**), and the data are representative of two independent experiments (**A, C–G**). ns, not significant; **P < 0.01, ***P < 0.001, ****P < 0.0001 (two-way ANOVA [**A, C, E**] and one-way ANOVA [**D**]).

To further validate the antiviral efficacy of NVP-HSP990 against RV, Caco-2 cells were infected with either the RV Wa or SA11 strain at a range of MOIs from 0.001 to 10. Robust inhibition of RV replication was consistently observed across all MOIs tested at 24 h p.i. (Fig. 2A). Nevertheless, pre-treatment of Caco-2 cells with NVP-HSP990 prior to RV infection (−2 ~ −1 h p.i.) did not significantly interfere with the establishment of RV infection or subsequent viral replication (Fig. S3A through C; Fig. 2B and C). NVP-HSP990 only modestly (~1-fold) reduced the final level of RV replication when administered during the course of RV infection (−1 ~ 0 h p.i.), suggesting that it is not highly effective at blocking RV entry into host cells; on the contrary, NVP-HSP990 administration after RV infection and during the whole process of cultivation (0 ~ 20 h p.i.) resulted in a highly significant, nearly 100-fold inhibitory effect, and exhibited a synergistic effect with NVP-HSP990 administration during the course of RV infection (−1 ~ 0 h p.i.) (Fig. 2B and C). Accordingly, the antiviral efficacy of NVP-HSP990 is governed by exposure duration, with longer exposure producing progressively greater suppression of RV replication (Fig. 2D).

qPCR analysis revealed a significant reduction of RNA synthesis of RV proteins VP2, VP6, NSP4, and NSP5 in RV-infected Caco-2 cells cultured with NVP-HSP990 (Fig. 2E), indicating that NVP-HSP990 significantly inhibited the transcription of the RV genome. Accordingly, RV structural proteins VP6 was remarkably reduced in RV-infected Caco-2 cells treated with NVP-HSP990 (Fig. 2F). IFA also showed potent inhibition of RV VP6 expression in RV-infected Caco-2 cells when treated with 100 nM NVP-HSP990 (Fig. 2G).

### NVP-HSP990 alters life state of host cells

RNA-seq analysis was performed to evaluate the impact of NVP-HSP990 treatment on the host cell transcriptome during RV infection. Principal component analysis revealed that both RV infection and NVP-HSP990 treatment were key factors influencing host gene expression patterns (Fig. S4A). As anticipated, RV infection triggered substantial transcriptional changes in host cells; however, NVP-HSP990 addition mitigated these changes (Fig. S4B). RV infection induced transcriptional modulations across various KEGG pathways; however, NVP-HSP990 effectively reversed these changes except for specific pathways like the IL-17 and TNF signaling pathways, which are associated with innate immunity or inflammation (Fig. S4C through F).

**Fig 3:**
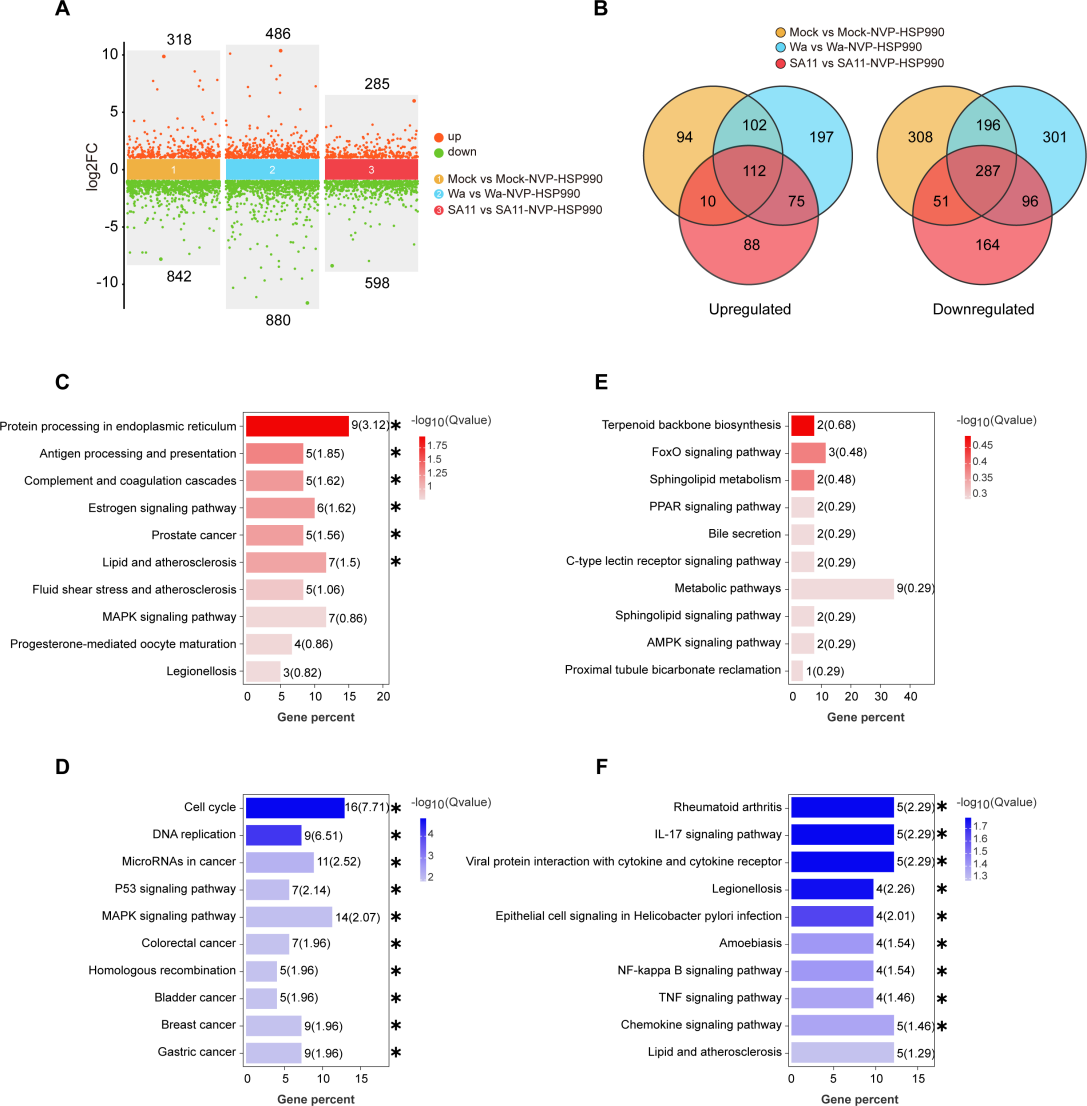
NVP-HSP990 alters the life state of host cells. Caco-2 cells were mock-infected with PBS or infected with RV Wa or SA11 strains (MOI = 3) and further cultivated with DMEM containing 100 nM NVP-HSP990 or an equal volume of DMSO as a control for 24 h. Then, the infected cells were harvested for RNA-seq analysis. (**A**) Multiple differential scatter plots of compared groups. (**B**) Venn diagrams of up- and downregulated genes between compared groups. (**C, D**) Top 10 (ranked by descending Q value) upregulated (**C**) and downregulated (**D**) KEGG pathways in all mock-, Wa-, and SA11-infected Caco-2 cells. (**E, F**) Top 10 (ranked by descending Q value) upregulated (**E**) and downregulated (**F**) KEGG pathways in both Wa- and SA11-infected but not in mock-infected Caco-2 cells. *Q value <0.05.

Next, we focused on the direct effects of NVP-HSP990 on host cells. We found that NVP-HSP990 induced significant transcriptional alterations in various genes, regardless of whether the host cells were mock-infected or RV-infected (Fig. 3A). These alterations were primarily associated with cell cycle, DNA replication, and various cancer-, signaling-, or metabolism-related pathways in host cells (Fig. S5A). Specifically, among these genes, 112 were upregulated and 287 were downregulated by NVP-HSP990 across all mock-, Wa-, and SA11-infected host cells (Fig. 3B; Tables S3 and S4). The upregulated genes were significantly enriched (Q value <0.05) in 6 KEGG pathways such as protein processing in the endoplasmic reticulum and antigen processing and presentation, while the downregulated genes were significantly enriched (Q value <0.05) in 14 KEGG pathways including cell cycle, DNA replication, MAPK signaling pathway, and various cancer-related pathways (Fig. 3C and D). Notably, 75 genes were upregulated and 96 genes were downregulated by NVP-HSP990 in both Wa- and SA11-infected host cells but not in mock-infected cells (Fig. 3B; Tables S5 and S6). These upregulated genes were nominally enriched (Q value >0.05) in metabolic processes, and the downregulated genes were significantly enriched (Q value <0.05) in nine KEGG pathways including inflammatory responses (Fig. 3E and F). These findings revealed a selective regulatory pattern, characterized by suppression of signaling and inflammation-related pathways, through which NVP-HSP990 mitigated the perturbations induced by RV in host cells.

### NVP-HSP990 modulated MAPK signaling pathway and mitigated disruption of tight junctions in RV infection

**Fig 4:**
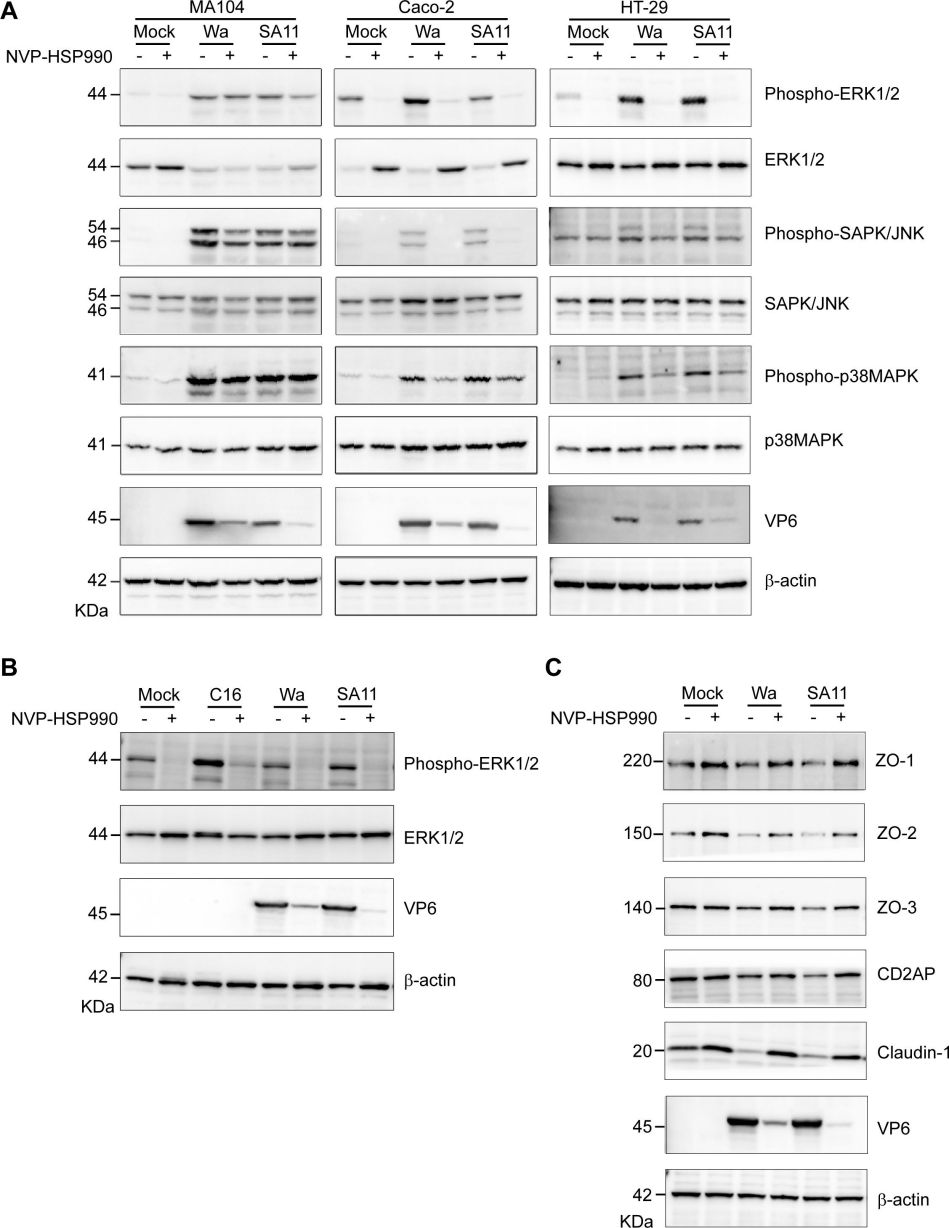
NVP-HSP990 inhibited MAPK activation and facilitated expression of tight junction-associated proteins in intestinal cells. (**A**) MA104, Caco-2, and HT-29 cells were mock-infected with PBS or infected with RV Wa or SA11 strains (MOI = 3), followed by treatment of 100 nM HSP990 (+) or an equal volume of DMSO as a control (−) for 20 h. Then the infected cells were harvested for WB analysis of MAPK components. (**B**) Caco-2 cells were mock-infected with PBS, treated with 1 µM C16-PAF(C16), or infected with RV Wa or SA11 strains (MOI = 3), and then treated with 100 nM HSP990 (+) or DMSO as a control (−) for 20 h. The infected cells were harvested for WB analysis of MAPK components. (**C**) Caco-2 cells were mock-infected with PBS or infected with RV Wa or SA11 strains (MOI = 3), and then treated with 100 nM HSP990 (+) or DMSO as control (−) for 20 h. Then the infected cells were harvested for WB analysis of tight junction-associated proteins. Data are representative of three (**A**) and two (**B and C**) independent experiments.

MAPK signaling pathway is mainly composed of ERK, JNK, and p38 signals and plays important roles in viral infections and host antiviral immunity (28, 29). In fact, the MAPK signaling pathway is reported to be activated and critical for RV replication (20, 30, 31). We found that NVP-HSP990 significantly upregulated some but downregulated more MAPK-related genes (Fig. 3C and D; Fig. S5B). The upregulated MAPK-related genes included some heat shock proteins (HSPB1, HSPA1A, HSPA1B, and so on) and some cytokines/cytokine receptors (such as PGF, PDGFRA, and PDGFRB), while the downregulated genes included JUN, JUND, MYC, DUSP2, DUSP5, and so on, which are involved in signal transduction (Fig. S5B and S6, Tables S7 to S9). These results indicated that NVP-HSP990 mainly inhibited MAPK signaling pathway in RV infection. Notably, NVP-HSP990 significantly inhibited the MAPK activation (especially ERK1/2 and p38 MAPK) in Caco-2 and HT-29 but not MA104 cells (Fig. 4A; Fig. S7). These results indicated that NVP-HSP990 might specifically inhibit the MAPK activation in intestinal epithelial cells, which are natural target cells of RV, thus favoring its anti-RV effect *in vivo*. Moreover, NVP-HSP990 inhibition of MAPK signaling pathway is not dependent on the inhibition of RV replication, because it also potently inhibited MAPK activation caused by non-infectious factors such as drug stimulation (Fig. 4B).

**Fig 5:**
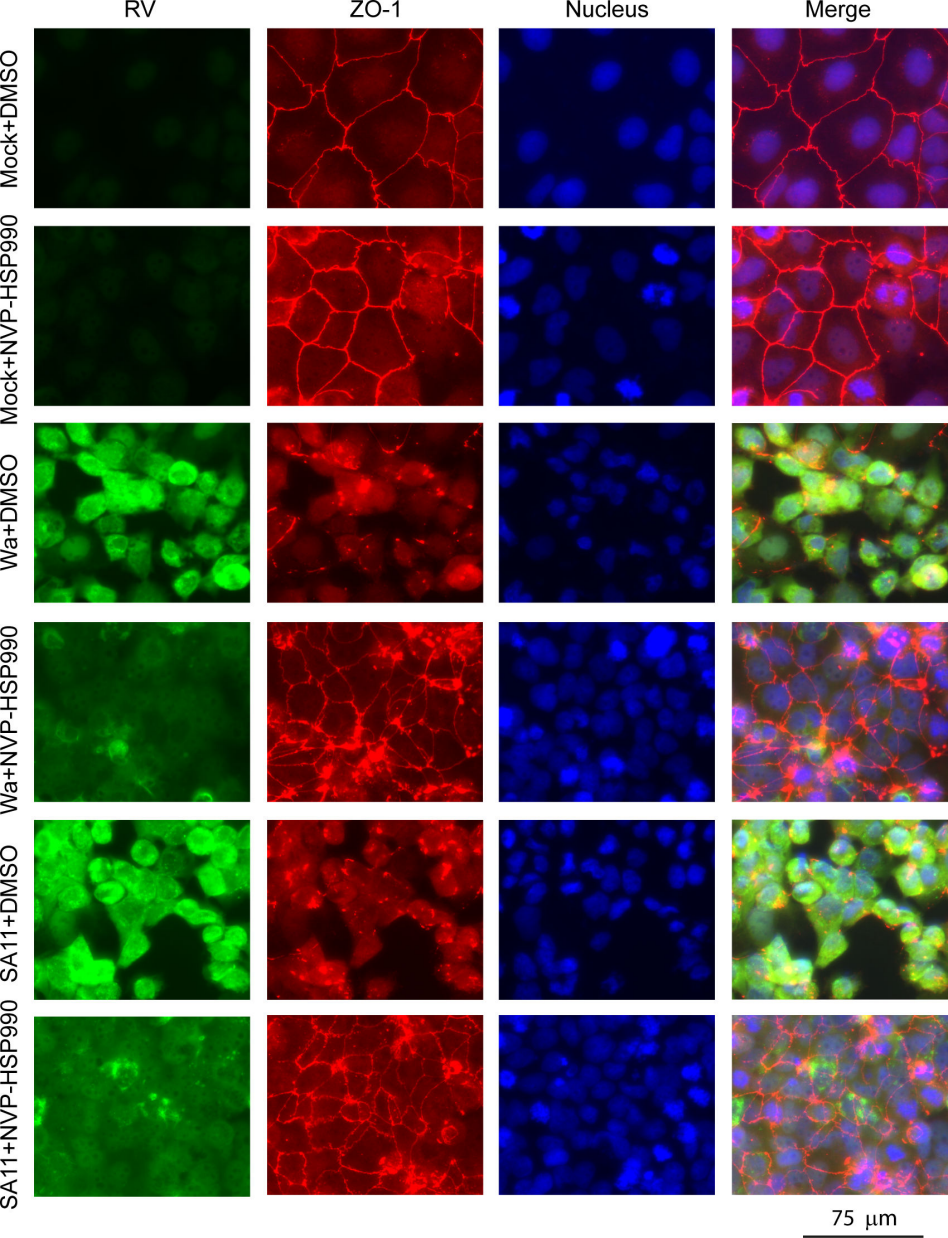
NVP-HSP990 mitigated disruption of tight junctions in RV infection. Caco-2 cells growing on coverslips were mock-infected or infected with RV Wa or SA11 strains (MOI = 3), and cultivated with DMEM containing 100 nM NVP-HSP990 or an equal volume of DMSO as a control after infection for another 18 h. Then the infected cells were applied for immunostaining for RV antigens (green), ZO-1 (red), and DAPI staining of nucleus (blue). Data are representative of two independent experiments.

Tight junctions are crucial for the survival and function of mature intestinal epithelial cells, and destruction of tight junctions causes intestinal inflammation and disorders (32, 33). Formation of tight junctions is modulated by intracellular signaling pathways including MAPK (34, 35). As NVP-HSP990 significantly inhibited the MAPK signaling pathway in intestinal cells, we wondered whether NVP-HSP990 also effectively mitigates RV-induced disruption of tight junctions. We found that NVP-HSP990 facilitated the expression of tight junction-associated proteins such as ZO-1, ZO-2, and claudin-1 (Fig. 4C). More importantly, NVP-HSP990 effectively restored structural disruption of tight junctions in RV infection (Fig. 5). Therefore, this function of NVP-HSP990 would benefit the protection of intestinal epithelium integrity.

### NVP-HSP990 alleviated RV infection in suckling mice

**Fig 6:**
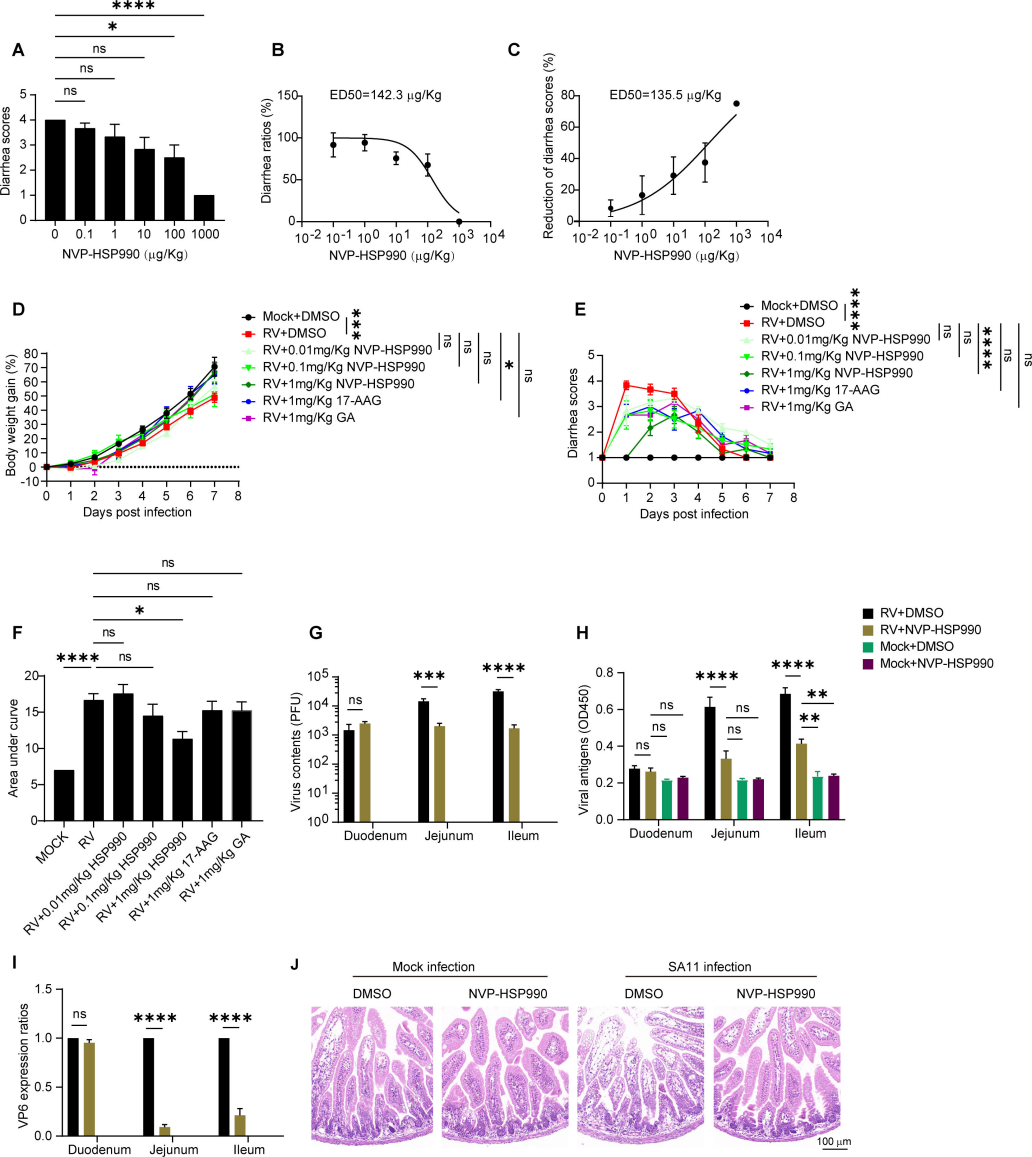
NVP-HSP990 inhibited RV infection in suckling mice. (**A–C**) Seven-day-old BALB/c suckling mice were orally infected with 1 × 10^6^ PFU of RV SA11 strain. At 2 h p.i., the mice were orally treated with indicated doses of drugs (*n* = 6 mice/group). Diarrhea scores (**A**), ED_50_ values of NVP-HSP990 to diarrhea occurrence (**B**), and for diarrhea score reduction (**C**) at 24 h p.i. are shown. (**D–F**) 7-day-old BALB/c suckling mice were orally infected with 1 × 10^6^ PFU of RV SA11 strain or mock-infected with PBS. At 2 h p.i., the mice were orally treated with indicated doses of drugs or equal volume of DMSO (*n* = 6 mice/group). Body weight gains (**D**) and diarrhea scores (**E**) of the suckling mice were monitored from 0 to 7 days post-infection, and area under the curve of diarrhea scores in each group was calculated (**F**). (**G–J**) Seven-day-old BALB/c suckling mice were orally inoculated with 1 × 10^6^ PFU of RV SA11 strain or mock-infected with PBS. At 2 h p.i., the mice were orally treated with 1 mg/kg of HSP990 with DMSO as control (*n* = 3 mice/group). At 16 h p.i., the infectious RV particles and RV antigens in contents of intestines were analyzed with PFA (**G**) and ELISA (**H**), respectively, RV VP6 transcription in intestinal epithelial cells was analyzed with qPCR (**I**), and the ileum of the mice was subjected to histopathological analysis with hematoxylin/eosin staining (**J**). Data are presented as mean ± SEM (**A, D–I**) and are representative of two independent experiments. ns, not significant; *P < 0.05, **P < 0.01, ***P < 0.001, ****P < 0.0001 (one-way ANOVA [**A and F**], two-way ANOVA [**D, E, G–I**]).

As NVP-HSP990 possessed potent anti-RV effects *in vitro* as shown above, we wondered whether it was effective for RV control *in vivo*. To this end, a series of doses of NVP-HSP990 were orally administered to suckling mice infected with the RV SA11 strain, and the diarrhea scores were evaluated daily. We found that the average diarrhea scores at 24 h p.i. (day 1) decreased along with the increase of NVP-HSP990 doses, and the reduction was especially remarkable at 1,000 µg/kg (Fig. 6A). NVP-HSP990 inhibited RV diarrhea occurrence with an ED_50_ (50% effective dose) value of 142.3 µg/kg, and it reduced diarrhea score with an ED_50_ value of 135.5 µg/kg (Fig. 6B and C). Treatment with 1 mg/kg NVP-HSP990 (once) did not hinder body growth of RV-infected suckling mice but significantly alleviated their diarrhea (Fig. 6D through F). Accordingly, infectious RV particles and viral antigens in intestinal contents, as well as transcription of RV VP6 protein in intestinal epithelial cells, were remarkably reduced in jejunum and ileum by administration of NVP-HSP990 compared to controls, though there were no significant differences in duodenum (Fig. 6G through I). We next assessed the protective effect of NVP-HSP990 against RV-induced ileum lesions. NVP-HSP990 treatment alone produced no appreciable pathological changes in the ileum of mock-infected mice. In contrast, RV infection induced pronounced foamy degeneration in the ileal epithelium. This pathological change was markedly attenuated by administration of NVP-HSP990 (Fig. 6J). These findings demonstrate that NVP-HSP990 can effectively alleviate RV infection *in vivo*.

**Fig 7:**
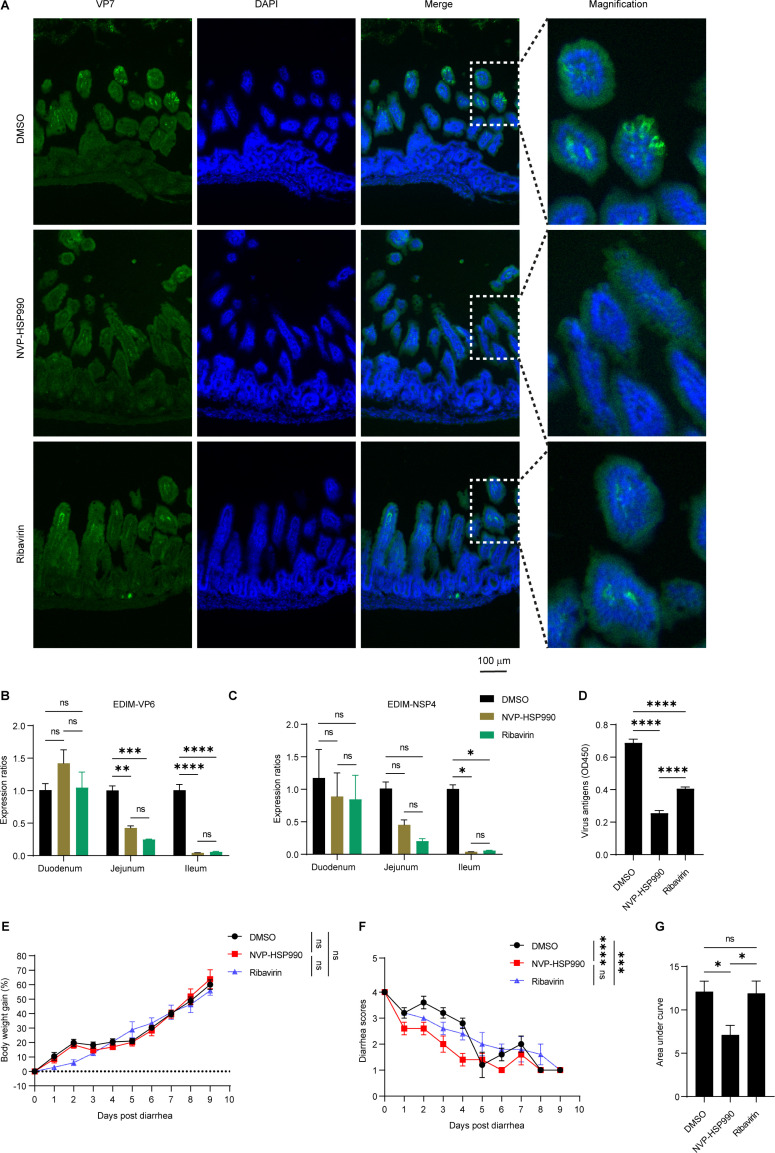
NVP-HSP990 exhibited therapeutic efficacy to RV infection. Seven-day-old BALB/c suckling mice were orally inoculated with 10× DD_50_ of RV EDIM strain. At 2 d p.i. when diarrhea occurred in all mice, the mice were orally treated with 0.2 mg/kg/day NVP-HSP990, 30 mg/kg/day ribavirin, or equal amount of DMSO as control. (**A–D**) After 3 days of drug treatment, mice intestines (ileum) were subjected to immunohistochemistry analysis of EDIM VP7 (green) and DAPI staining of nucleus (blue) (**A**), transcription of EDIM VP6 (**B**) and NSP4 (**C**) in intestinal epithelial cells was analyzed with qPCR, and viral antigens in the colon were analyzed by ELISA (**D**) (*n* = 3 mice/group). (**E–G**) The EDIM-infected suckling mice with diarrhea were treated with the drugs as above for 5 days and then observed for an additional 5 days (*n* = 5 mice/group). Body weight gains (**E**) and diarrhea scores (**F**) of the suckling mice were monitored, and area under the curve of diarrhea scores in each group was calculated (**G**). Data are presented as mean ± SEM (**B–G**) and are representative of two independent experiments. ns, not significant, *P < 0.05, **P < 0.01, ***P < 0.001, ****P < 0.0001 (one-way ANOVA [**D, G**], two-way ANOVA [**B, C, E, F**]).

Furthermore, we evaluated the therapeutic efficacy of NVP-HSP990 by administering it orally to suckling mice after diarrhea onset following infection with the mouse-derived RV strain EDIM. After 3 consecutive days of treatment, histopathological and immunohistochemical analyses revealed a marked reduction in both foamy degeneration and viral infection in the small intestinal epithelial cells of the NVP-HSP990-treated group compared to the untreated controls. However, no appreciable difference in the extent of infection or tissue damage was observed between the NVP-HSP990- and ribavirin-treated groups (Fig. S8 and S9; Fig. 7A). Viral antigen (VP6 and NSP4) expression in intestinal epithelial cells was significantly reduced in the NVP-HSP990 group compared to the untreated group, and this reduction was comparable to that achieved by the conventional antiviral drug ribavirin, although no significant differences were observed in the duodenum among the groups (Fig. 7B and C). Additionally, RV viral antigens in colonic contents were significantly lower in the NVP-HSP990 group than in either the untreated or ribavirin-treated groups (Fig. 7D).

To evaluate the longer therapeutic effect of NVP-HSP990 on RV diarrhea, suckling mice were treated with the drugs continuously for 5 days after the onset of diarrhea, and their body weights and diarrhea scores were monitored during the process and for another 5 days after drug administration. The results showed that NVP-HSP990 did not adversely affect weight gain in EDIM-infected suckling mice (Fig. 7E). However, NVP-HSP990 significantly reduced diarrhea scores compared to the untreated group, and it also demonstrated a superior therapeutic effect to ribavirin (Fig. 7F and G).

## DISCUSSION

Cell stress induced by viral infection results in enhanced expression of various heat shock proteins, including HSP40, HSP70, HSP90, and so on, and viral replication is directly or indirectly dependent on one or more of these HSPs (36, 37). Many viruses, including RV, require the participation of HSP90 in their replication (12–14, 19, 38, 39), so it sounds promising to develop broad-spectrum, HSP90-targeting antiviral drugs, which would be less affected by viral mutations and more stable in antiviral effect. Therefore, small-molecule inhibitors of HSP90 are currently popular in antiviral drug discovery (21, 40–42). Unfortunately, considering their unsatisfying antiviral efficacy and toxicity, there have been no HSP90 inhibitors in clinical use for viral control until now. The design of small-molecule HSP90 inhibitors is commonly based on the inhibitory mechanism of the natural HSP90 inhibitor GA or Radicicol, which targets the ATP pocket of N-terminal domain of HSP90 to block the ATPase activity. With the increasing understanding of HSP90 subtypes and the interactions between HSP90 and its client proteins, a series of novel HSP90 inhibitors have emerged, which selectively inhibit HSP90 subtypes or specifically block the interaction between HSP90 and client proteins, so as to improve their inhibitory efficacy and reduce their cytotoxicity (43, 44). NVP-HSP990 is much smaller than and structurally distinct from GA and 17-AAG, and it targets the N-terminals of both HSP90α and HSP90β with very high specificity (IC_50_: 0.6 nM and 0.8 nM, respectively). NVP-HSP990 is orally bioavailable and brain-penetrating and has been tested in the treatment of various tumors and Huntington’s disease (26, 45–47). However, there have been few reports on the role of NVP-HSP990 in controlling viral infections, though a recent *in silico* study predicted that NVP-HSP990 might alleviate COVID-19 symptoms through anti-inflammatory effects on SARS-CoV-2-infected lung cells (48). In this study, we showed that NVP-HSP990 inhibited RV infection with high SI *in vitro* and effectively alleviated RV diarrhea in suckling mice. Our findings suggest that NVP-HSP990 may be a promising antiviral drug candidate for RV infection, and that targeting HSP90 should remain to be a promising strategy for antiviral drug development.

Viral infection usually triggers the modulation of host signaling pathways, resulting in transcriptional changes of various host genes to remodel host life systems for better viral replication (49–51). On the other hand, viral infection inevitably triggers the activation of immunity/inflammation-associated signaling pathways for host antiviral innate immunity. In this study, although RV infection triggered significant alterations in host gene transcription across various signaling pathways, NVP-HSP990 effectively mitigated these impacts on host cells. Notably, the activation of immune/inflammatory signaling pathways (such as IL-17 and TNF signaling pathways) was still significant under NVP-HSP990 addition. These results indicate that NVP-HSP990 not only potently inhibits RV replication but also preserves the host’s ability to mount an innate immune response and trigger inflammation. This dual action facilitates complete viral clearance and represents a significant advantage of NVP-HSP990 as a potential antiviral candidate.

Upon NVP-HSP990 treatment, the most affected pathways in Caco-2 cells included cell cycle, DNA repair, and cancer-related pathways, which is consistent with its role as an effective anti-tumor drug. To be noted, MAPK signaling pathway was negatively regulated by NVP-HSP990 in both non-infected and RV-infected Caco-2 cells, suggesting that MAPK signaling pathway is sensitive to HSP90 inhibition by NVP-HSP990, aligning with the report that proper folding of RAF kinases of the RAS-MAPK signaling pathway relies on the interaction with HSP90 (52). RV infection induces MAPK activation, including the activation of ERK, JNK, and p38, among which ERK and p38 are critical for RV replication, while JNK is critical for host IFN-β production (30, 31, 53, 54). In this study, we demonstrated that NVP-HSP990 potently suppressed the activation of ERK and p38 in RV-infected intestinal epithelial cells like Caco-2 and HT-29 but not in renal epithelial cells MA104, although all of them are susceptible to RV infection. Therefore, NVP-HSP990 might specifically inhibit MAPK activation in intestinal epithelial cells, which should facilitate its anti-RV efficacy *in vivo*. To be noted, NVP-HSP990 inhibition of the MAPK signaling pathway did not depend on RV inhibition, as it also inhibited the basal or drug-induced MAPK activation in intestinal epithelial cells.

Disruption of tight junctions leads to the necrosis or anoikis of mature intestinal epithelial cells, which is a key step in the pathogenesis of intestinal infectious diseases (55). Many studies show that activation of the p38 signaling pathway disrupts cellular tight junctions (35, 56). In this study, we found that RV infection activated p38 signaling and disrupted cellular tight junctions in Caco-2 cells, which were consistent with previous reports (31, 57). Nevertheless, NVP-HSP990 significantly inhibited p38 activation and mitigated tight junction disruption in RV-infected Caco-2 cells, endowing NVP-HSP990 with the function of protecting intestinal epithelium in RV infection. The blood-brain barrier (BBB), primarily composed of tight junctions between brain microvascular endothelial cells, plays a crucial role in preventing pathogens from entering the brain. Pathogens or toxins can invade the brain by disrupting tight junctions of brain microvascular endothelial cells, thereby increasing BBB permeability (58). Numerous reports demonstrate the presence of viral particles and antigens in the brain during RV infection (5), suggesting a potential compromise of BBB integrity. Therefore, the protective effect of NVP-HSP990 on tight junctions could mitigate BBB disruption during RV infection, potentially lowering the risk of encephalopathy development.

Besides gastrointestinal symptoms such as diarrhea and vomiting, severe RV infections often affect important organs such as the brain, heart, kidneys, and liver; for example, RV infection usually causes epileptic seizures in children, although the mechanism is currently unclear (5). Conventional antiviral drugs such as nucleoside analogs and type I interferon are usually not included in the treatment of RV diarrhea due to adverse side effects (8, 9), thus symptomatic treatment is usually the main choice, which sometimes leads to systemic RV infection and even mortality due to persistent infection. Therefore, the treatment of RV infection should not merely focus on symptomatic treatment; timely antiviral treatment shall be important to reduce complications and mortality. Here, we demonstrated that besides potently inhibiting RV infection *in vitro*, NVP-HSP990 also effectively suppressed intestinal RV infection and alleviated diarrhea in BALB/c suckling mice, rendering it a promising candidate for an anti-RV drug. In addition, given that NVP-HSP990 is able to penetrate BBB and very low dose (0.1 mg/kg–0.2 mg/kg) of NVP-HSP990 shows potent antiepileptic activity (59, 60), it could also represent a promising therapeutic strategy for RV encephalopathy whether mediated by direct RV neuroinvasion or potential unknown mechanisms.

Admittedly, in many regions where rotavirus remains a significant issue, clinical diagnostic tests are often unavailable, limiting the ability to confirm rotavirus as the cause of acute gastroenteritis. Moreover, rotavirus infections are typically acute and self-limiting (61), which complicates the use of antiviral treatments. However, the discovery of a potent antiviral compound like NVP-HSP990 remains highly valuable, as it opens the door to exploring more targeted clinical applications. Firstly, NVP-HSP990 could be particularly beneficial in chronic infection scenarios. For example, in immunocompromised individuals—such as those undergoing chemotherapy, organ transplant recipients, or those with primary immunodeficiencies—rotavirus infections can lead to severe, protracted enteritis that may become life-threatening (62). In these cases of chronic or persistent infection, a potent oral antiviral like NVP-HSP990 could offer a critical treatment option. Secondly, severe acute rotavirus infections can sometimes lead to multi-organ complications and be fatal (63). Timely administration of antiviral drugs may help alleviate the severity of intestinal infections and inhibit possible extraintestinal spread, thus reducing the risk of mortality and associated complications. Additionally, future research could investigate NVP-HSP990’s potential as post-exposure prophylaxis, particularly for controlling outbreaks in closed environments such as pediatric wards or childcare centers.

### Conclusion

**Fig 8:**
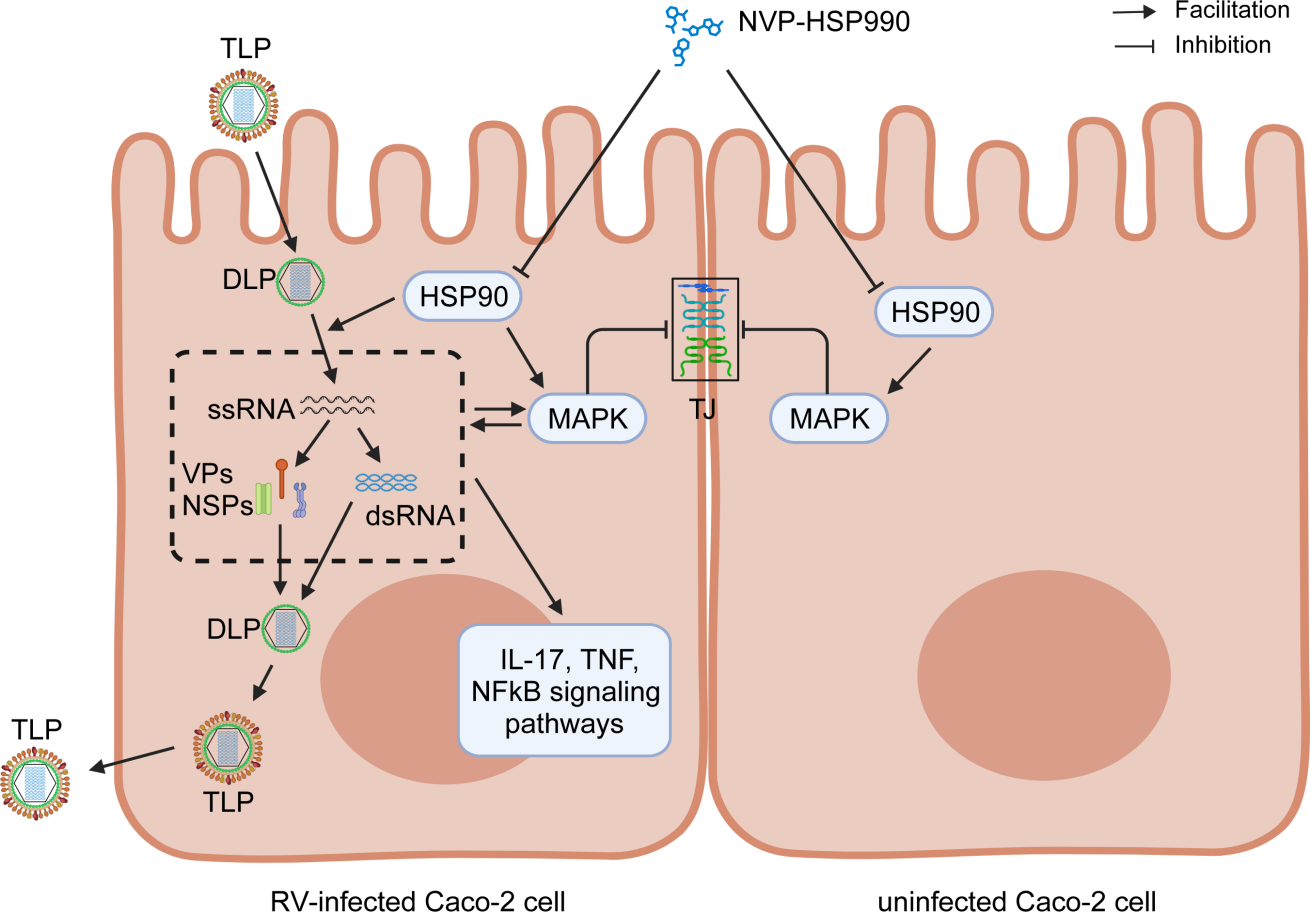
The mechanisms underlying RV inhibition and epithelial protection by NVP-HSP990.

In this study, we demonstrated that the small-molecule HSP990 inhibitor NVP-HSP990 robustly blocked RV replication with low cytotoxicity *in vitro*, mitigated RV impacts on the host transcriptome, and repressed RV-induced MAPK activation and tight junction disruption in intestinal cells (Fig. 8), which finally contributed to effective alleviation of RV diarrhea in suckling mice. These findings, coupled with excellent oral bioavailability and brain-penetrating ability, make NVP-HSP990 a novel, promising candidate antiviral drug for alleviating RV infection.

## Data Availability

All the data and materials associated with this study are available upon request. The raw RNA-seq data reported in this paper have been deposited in the Genome Sequence Archive (GSA) of the National Genomics Data Center, China National Center for Bioinformation, under the accession number HRA013787.

## SUPPLEMENTAL MATERIAL

Text S1 jvi.01883-25-s0001.docx Supplemental methods; Fig. S1 to S9.

Text S2 jvi.01883-25-s0002.docx Antibodies and reagents.

Table S1 jvi.01883-25-s0003.docx Primers used in qPCR analysis.

Table S2 jvi.01883-25-s0004.docx CC50, IC50, and SI values.

Tables S3 to S9 jvi.01883-25-s0005.xls Genes that were upregulated, downregulated, and significantly modified under various conditions.

ASM does not own the copyrights to Supplemental Material that may be linked to, or accessed through, an article. The authors have granted ASM a non-exclusive, world-wide license to publish the Supplemental Material files. Please contact the corresponding author directly for reuse.

## References

[1] Matthijnssens J, Attoui H, Bányai K, Brussaard CPD, Danthi P, del Vas M, Dermody TS, Duncan R, Fāng Q, Johne R, Mertens PPC, Mohd Jaafar F, Patton JT, Sasaya T, Suzuki N, Wei T. 2022. ICTV virus taxonomy profile: Spinareoviridae 2022. J Gen Virol 103. doi:10.1099/jgv.0.001781 PMC12642366 36394457

[2] Hasan H, Nasirudeen NA, Ruzlan MAF, Mohd Jamil MA, Ismail NAS, Wahab AA, Ali A. 2021. Acute infectious gastroenteritis: the causative agents, omics-based detection of antigens and novel biomarkers. Children (Basel) 8:1112. doi:10.3390/children8121112 34943308 PMC8700514

[3] Hellysaz A, Hagbom M. 2021. Understanding the central nervous system symptoms of rotavirus: a qualitative review. Viruses 13:658. doi:10.3390/v13040658 33920421 PMC8069368

[4] Lorrot M, Vasseur M. 2007. How do the rotavirus NSP4 and bacterial enterotoxins lead differently to diarrhea? Virol J 4:31. doi:10.1186/1743-422X-4-31 17376232 PMC1839081

[5] Dian Z, Sun Y, Zhang G, Xu Y, Fan X, Yang X, Pan Q, Peppelenbosch M, Miao Z. 2021. Rotavirus-related systemic diseases: clinical manifestation, evidence and pathogenesis. Crit Rev Microbiol 47:580–595. doi:10.1080/1040841X.2021.1907738 33822674

[6] Cates JE, Tate JE, Parashar U. 2022. Rotavirus vaccines: progress and new developments. Expert Opin Biol Ther 22:423–432. doi:10.1080/14712598.2021.1977279 34482790 PMC10839819

[7] Cohen AL, Platts-Mills JA, Nakamura T, Operario DJ, Antoni S, Mwenda JM, Weldegebriel G, Rey-Benito G, de Oliveira LH, Ortiz C, et al.. 2022. Aetiology and incidence of diarrhoea requiring hospitalisation in children under 5 years of age in 28 low-income and middle-income countries: findings from the global pediatric diarrhea surveillance network. BMJ Glob Health 7:e009548. doi:10.1136/bmjgh-2022-009548 PMC9445824 36660904

[8] Sun S, Lin X, Yang Y, Cen J, Luo F, Chen X. 2020. Acupoint application for rotavirus diarrhea in infants and children: a protocol for systematic review and meta analysis. Medicine (Baltimore) 99:e22227. doi:10.1097/MD.0000000000022227 32957362 PMC7505364

[9] Jiang L, Tang A, Song L, Tong Y, Fan H. 2023. Advances in the development of antivirals for rotavirus infection. Front Immunol 14:1041149. doi:10.3389/fimmu.2023.1041149 37006293 PMC10063883

[10] Hoter A, El-Sabban ME, Naim HY. 2018. The HSP90 family: structure, regulation, function, and implications in health and disease. Int J Mol Sci 19:2560. doi:10.3390/ijms19092560 30158430 PMC6164434

[11] Powers MV, Workman P. 2006. Targeting of multiple signalling pathways by heat shock protein 90 molecular chaperone inhibitors. Endocr Relat Cancer 13 Suppl 1:S125–35. doi:10.1677/erc.1.01324 17259553

[12] Hu J, Seeger C. 1996. Hsp90 is required for the activity of a hepatitis B virus reverse transcriptase. Proc Natl Acad Sci USA 93:1060–1064. doi:10.1073/pnas.93.3.1060 8577714 PMC40030

[13] Okamoto T, Nishimura Y, Ichimura T, Suzuki K, Miyamura T, Suzuki T, Moriishi K, Matsuura Y. 2006. Hepatitis C virus RNA replication is regulated by FKBP8 and Hsp90. EMBO J 25:5015–5025. doi:10.1038/sj.emboj.7601367 17024179 PMC1618089

[14] Naito T, Momose F, Kawaguchi A, Nagata K. 2007. Involvement of Hsp90 in assembly and nuclear import of influenza virus RNA polymerase subunits. J Virol 81:1339–1349. doi:10.1128/JVI.01917-06 17121807 PMC1797515

[15] Yadav M, Singh AK, Kumar A, Thareja S, Kumar P. 2022. An insight to heat shock protein 90: a remedy for multiple problems. CPD 28:2664–2676. doi:10.2174/1381612828666220829120630 36043709

[16] Rico J, Perez C, Hernandez J, Guerrero C, Acosta O. 2021. Cell surface heat shock protein-mediated entry of tumor cell-adapted rotavirus into U-937 cells. Folia Microbiol (Praha) 66:623–638. doi:10.1007/s12223-020-00845-x 33950511

[17] Pérez C, Rico J, Guerrero CA, Acosta O. 2021. Role of heat-shock proteins in infection of human adenocarcinoma cell line MCF-7 by tumor-adapted rotavirus isolates. Colomb Med 52:e2024196. doi:10.25100/cm.v52i1.4196 PMC8054709 33911319

[18] Rico J, Perez C, Guerrero R, Hernandez J, Guerrero C, Acosta O. 2020. Implication of heat shock proteins in rotavirus entry into Reh cells. Acta Virol 64:433–450. doi:10.4149/av_2020_406 33112641

[19] Dutta D, Bagchi P, Chatterjee A, Nayak MK, Mukherjee A, Chattopadhyay S, Nagashima S, Kobayashi N, Komoto S, Taniguchi K, Chawla-Sarkar M. 2009. The molecular chaperone heat shock protein-90 positively regulates rotavirus infectionx. Virology (Auckl) 391:325–333. doi:10.1016/j.virol.2009.06.044 19628238

[20] Huang H, Liao D, Zhou G, Zhu Z, Cui Y, Pu R. 2020. Antiviral activities of resveratrol against rotavirus in vitro and in vivo. Phytomedicine 77:153230. doi:10.1016/j.phymed.2020.153230 32682225

[21] Wang Y, Jin F, Wang R, Li F, Wu Y, Kitazato K, Wang Y. 2017. HSP90: a promising broad-spectrum antiviral drug target. Arch Virol 162:3269–3282. doi:10.1007/s00705-017-3511-1 28780632

[22] Xie X, Zhang N, Li X, Huang H, Peng C, Huang W, Foster LJ, He G, Han B. 2023. Small-molecule dual inhibitors targeting heat shock protein 90 for cancer targeted therapy. Bioorg Chem 139:106721. doi:10.1016/j.bioorg.2023.106721 37467620

[23] Gorska M, Popowska U, Sielicka-Dudzin A, Kuban-Jankowska A, Sawczuk W, Knap N, Cicero G, Wozniak F. 2012. Geldanamycin and its derivatives as Hsp90 inhibitors. Front Biosci 17:2269–2277. doi:10.2741/4050 22652777

[24] Kim T, Keum G, Pae AN. 2013. Discovery and development of heat shock protein 90 inhibitors as anticancer agents: a review of patented potent geldanamycin derivatives. Expert Opin Ther Pat 23:919–943. doi:10.1517/13543776.2013.780597 23641970

[25] McBride CM, Levine B, Xia Y, Bellamacina C, Machajewski T, Gao Z, Renhowe P, Antonios-McCrea W, Barsanti P, Brinner K, et al.. 2014. Design, structure-activity relationship, and in vivo characterization of the development candidate NVP-HSP990. J Med Chem 57:9124–9129. doi:10.1021/jm501107q 25368984

[26] Menezes DL, Taverna P, Jensen MR, Abrams T, Stuart D, Yu GK, Duhl D, Machajewski T, Sellers WR, Pryer NK, Gao Z. 2012. The novel oral Hsp90 inhibitor NVP-HSP990 exhibits potent and broad-spectrum antitumor activities in vitro and in vivo. Mol Cancer Ther 11:730–739. doi:10.1158/1535-7163.MCT-11-0667 22246440

[27] Boshuizen JA, Reimerink JHJ, Korteland-van Male AM, van Ham VJJ, Koopmans MPG, Büller HA, Dekker J, Einerhand AWC. 2003. Changes in small intestinal homeostasis, morphology, and gene expression during rotavirus infection of infant mice. J Virol 77:13005–13016. doi:10.1128/jvi.77.24.13005-13016.2003 14645557 PMC296055

[28] Chander Y, Kumar R, Khandelwal N, Singh N, Shringi BN, Barua S, Kumar N. 2021. Role of p38 mitogen-activated protein kinase signalling in virus replication and potential for developing broad spectrum antiviral drugs. Rev Med Virol 31:1–16. doi:10.1002/rmv.2217 33450133

[29] MacMicking JD. 2022. Rewiring the logic board of IFN signaling. Sci Signal 15:eadf0778. doi:10.1126/scisignal.adf0778 36512642

[30] Soliman M, Seo JY, Kim DS, Kim JY, Park JG, Alfajaro MM, Baek YB, Cho EH, Kwon J, Choi JS, Kang MI, Park SI, Cho KO. 2018. Activation of PI3K, Akt, and ERK during early rotavirus infection leads to V-ATPase-dependent endosomal acidification required for uncoating. PLoS Pathog 14:e1006820. doi:10.1371/journal.ppat.1006820 29352319 PMC5792019

[31] Holloway G, Coulson BS. 2006. Rotavirus activates JNK and p38 signaling pathways in intestinal cells, leading to AP-1-driven transcriptional responses and enhanced virus replication. J Virol 80:10624–10633. doi:10.1128/JVI.00390-06 16928761 PMC1641755

[32] Zihni C, Mills C, Matter K, Balda MS. 2016. Tight junctions: from simple barriers to multifunctional molecular gates. Nat Rev Mol Cell Biol 17:564–580. doi:10.1038/nrm.2016.80 27353478

[33] Furuse M, Takai Y. 2021. Recent advances in understanding tight junctions. Fac Rev 10:18. doi:10.12703/r/10-18 33718935 PMC7946388

[34] Rungrasameviriya P, Santilinon A, Atichartsintop P, Hadpech S, Thongboonkerd V. 2024. Tight junction and kidney stone disease. Tissue Barriers 12:2210051. doi:10.1080/21688370.2023.2210051 37162265 PMC10832927

[35] Cong X, Kong W. 2020. Endothelial tight junctions and their regulatory signaling pathways in vascular homeostasis and disease. Cell Signal 66:109485. doi:10.1016/j.cellsig.2019.109485 31770579

[36] Bolhassani A, Agi E. 2019. Heat shock proteins in infection. Clin Chim Acta 498:90–100. doi:10.1016/j.cca.2019.08.015 31437446

[37] Zhang X, Yu W. 2022. Heat shock proteins and viral infection. Front Immunol 13:947789. doi:10.3389/fimmu.2022.947789 35990630 PMC9389079

[38] Evers DL, Chao CF, Zhang Z, Huang ES. 2012. 17-allylamino-17-(demethoxy)geldanamycin (17-AAG) is a potent and effective inhibitor of human cytomegalovirus replication in primary fibroblast cells. Arch Virol 157:1971–1974. doi:10.1007/s00705-012-1379-7 22711259

[39] Wang C, Liu P, Luo J, Ding H, Gao Y, Sun L, Luo F, Liu X, He H. 2017. Geldanamycin reduces acute respiratory distress syndrome and promotes the survival of mice infected with the highly virulent H5N1 influenza virus. Front Cell Infect Microbiol 7:267. doi:10.3389/fcimb.2017.00267 28664154 PMC5471324

[40] Koren J 3rd, Blagg BSJ. 2020. The right tool for the job: an overview of Hsp90 inhibitors. Adv Exp Med Biol 1243:135–146. doi:10.1007/978-3-030-40204-4_9 32297216

[41] Serwetnyk MA, Blagg BSJ. 2021. The disruption of protein-protein interactions with co-chaperones and client substrates as a strategy towards Hsp90 inhibition. Acta Pharm Sin B 11:1446–1468. doi:10.1016/j.apsb.2020.11.015 34221862 PMC8245820

[42] Solit DB, Chiosis G. 2008. Development and application of Hsp90 inhibitors. Drug Discov Today 13:38–43. doi:10.1016/j.drudis.2007.10.007 18190862

[43] Costa TEMM, Raghavendra NM, Penido C. 2020. Natural heat shock protein 90 inhibitors in cancer and inflammation. Eur J Med Chem 189:112063. doi:10.1016/j.ejmech.2020.112063 31972392

[44] Li L, Wang L, You QD, Xu XL. 2020. Heat shock protein 90 inhibitors: an update on achievements, challenges, and future directions. J Med Chem 63:1798–1822. doi:10.1021/acs.jmedchem.9b00940 31663736

[45] Jackrel ME, Shorter J. 2011. Shock and awe: unleashing the heat shock response to treat huntington disease. J Clin Invest 121:2972–2975. doi:10.1172/JCI59190 21785212 PMC3148752

[46] Oikonomou A, Valsecchi L, Quadri M, Watrin T, Scharov K, Procopio S, Tu JW, Vogt M, Savino AM, Silvestri D, Valsecchi MG, Biondi A, Borkhardt A, Bhatia S, Cazzaniga G, Fazio G, Bardini M, Palmi C. 2023. High-throughput screening as a drug repurposing strategy for poor outcome subgroups of pediatric B-cell precursor acute lymphoblastic leukemia. Biochem Pharmacol 217:115809. doi:10.1016/j.bcp.2023.115809 37717691

[47] Spreafico A, Delord J-P, De Mattos-Arruda L, Berge Y, Rodon J, Cottura E, Bedard PL, Akimov M, Lu H, Pain S, Kaag A, Siu LL, Cortes J. 2015. A first-in-human phase I, dose-escalation, multicentre study of HSP990 administered orally in adult patients with advanced solid malignancies. Br J Cancer 112:650–659. doi:10.1038/bjc.2014.653 25625276 PMC4333497

[48] Oh KK, Adnan M, Cho DH. 2021. Drug-repurposing against COVID-19 by targeting a key signaling pathway: an in silico study. Med Hypotheses 155:110656. doi:10.1016/j.mehy.2021.110656 34399157 PMC8349734

[49] Pleschka S. 2008. RNA viruses and the mitogenic Raf/MEK/ERK signal transduction cascade. Biol Chem 389:1273–1282. doi:10.1515/BC.2008.145 18713014

[50] Wu Y, Zhang Z, Li Y, Li Y. 2021. The regulation of integrated stress response signaling pathway on viral infection and viral antagonism. Front Microbiol 12:814635. doi:10.3389/fmicb.2021.814635 35222313 PMC8874136

[51] Akbay B, Shmakova A, Vassetzky Y, Dokudovskaya S. 2020. Modulation of mTORC1 signaling pathway by HIV-1. Cells 9:1090. doi:10.3390/cells9051090 32354054 PMC7291251

[52] Mitra S, Ghosh B, Gayen N, Roy J, Mandal AK. 2016. Bipartite role of heat shock protein 90 (Hsp90) keeps CRAF kinase poised for activation. J Biol Chem 291:24579–24593. doi:10.1074/jbc.M116.746420 27703006 PMC5114410

[53] Jafri M, Donnelly B, McNeal M, Ward R, Tiao G. 2007. MAPK signaling contributes to rotaviral-induced cholangiocyte injury and viral replication. Surgery 142:192–201. doi:10.1016/j.surg.2007.03.008 17689685

[54] He H, Zhou D, Fan W, Fu X, Zhang J, Shen Z, Li J, Li J, Wu Y. 2012. Cyclophilin A inhibits rotavirus replication by facilitating host IFN-I production. Biochem Biophys Res Commun 422:664–669. doi:10.1016/j.bbrc.2012.05.050 22609402

[55] Paradis T, Bègue H, Basmaciyan L, Dalle F, Bon F. 2021. Tight junctions as a key for pathogens invasion in intestinal epithelial cells. Int J Mol Sci 22:2506. doi:10.3390/ijms22052506 33801524 PMC7958858

[56] Kang J, Zhou Y, Zhu C, Ren T, Zhang Y, Xiao L, Fang B. 2022. Ginsenoside Rg1 mitigates porcine intestinal tight junction disruptions induced by LPS through the p38 MAPK/NLRP3 inflammasome pathway. Toxics 10:285. doi:10.3390/toxics10060285 35736894 PMC9228030

[57] Obert G, Peiffer I, Servin AL. 2000. Rotavirus-induced structural and functional alterations in tight junctions of polarized intestinal Caco-2 cell monolayers. J Virol 74:4645–4651. doi:10.1128/jvi.74.10.4645-4651.2000 10775600 PMC111984

[58] Wei C, Jiang W, Wang R, Zhong H, He H, Gao X, Zhong S, Yu F, Guo Q, Zhang L, Schiffelers LDJ, Zhou B, Trepel M, Schmidt FI, Luo M, Shao F. 2024. Brain endothelial GSDMD activation mediates inflammatory BBB breakdown. Nature 629:893–900. doi:10.1038/s41586-024-07314-2 38632402

[59] Sha L, Chen T, Deng Y, Du T, Ma K, Zhu W, Shen Y, Xu Q. 2020. Hsp90 inhibitor HSP990 in very low dose upregulates EAAT2 and exerts potent antiepileptic activity. Theranostics 10:8415–8429. doi:10.7150/thno.44721 32724478 PMC7381737

[60] Labbadia J, Cunliffe H, Weiss A, Katsyuba E, Sathasivam K, Seredenina T, Woodman B, Moussaoui S, Frentzel S, Luthi-Carter R, Paganetti P, Bates GP. 2011. Altered chromatin architecture underlies progressive impairment of the heat shock response in mouse models of Huntington disease. J Clin Invest 121:3306–3319. doi:10.1172/JCI57413 21785217 PMC3148745

[61] Parashar UD, Hummelman EG, Bresee JS, Miller MA, Glass RI. 2003. Global illness and deaths caused by rotavirus disease in children. Emerg Infect Dis 9:565–572. doi:10.3201/eid0905.020562 12737740 PMC2972763

[62] Ribeiro J, Ferreira D, Arrabalde C, Almeida S, Baldaque I, Sousa H. 2015. Prevalence of adenovirus and rotavirus infection in immunocompromised patients with acute gastroenteritis in Portugal. World J Virol 4:372–376. doi:10.5501/wjv.v4.i4.372 26568919 PMC4641229

[63] Tarris G, Belliot G, Callier P, Huet F, Martin L, de Rougemont A. 2019. Pathology of rotavirus-driven multiple organ failure in a 16-month-old boy. Pediatr Infect Dis J 38:e326–e328. doi:10.1097/INF.0000000000002472 31634298

