# Supplementary material for: A small-molecule HSP90 inhibitor, NVP-HSP990, alleviates rotavirus infection
Source: J Virol. 2025 Dec 10;100(1):e01883-25. doi: 10.1128/jvi.01883-25 (PMC12817916; doi:10.1128/jvi.01883-25)
Supplement: Text S1 — Supplemental methods; Fig. S1 to S9. [file jvi.01883-25-s0001.docx]

**Supplementary Materials and Methods**

*Virus preparation and titration*

RV (Wa, SA11, and EDIM) preparation was according to previously reported methods with some modifications (1, 2). Briefly, Wa/SA11-infected MA104 cells and the culture medium were frozen/thawed twice and centrifuged at 10,000 g for 20 min at 4°C for debris clearance. Supernatant of Wa strain was concentrated by centrifugation at 100,000 g with a SW28 rotor for 2 h at 4°C. For concentration of SA11 strain, NaCl was added to the supernatant of SA11 strain to a final concentration of 0.5 M, then Polyethylene glycol (PEG, MW6,000) (Sangon Biotech, China) was added to a final concentration of 8% (w/v) with stirring overnight at 4°C, followed with centrifugation at 10,000 g for 2 h at 4°C. Both the virus pellets were suspended in TNC buffer (50 mmol/L Tris, 140 mmol/L NaCl, 10 mmol/L CaCl_2_, pH 8.0), filtered with 0.22 μm filters and subjected to sucrose density gradient centrifugation. Wa/SA11 strains were titrated by plaque formation assay (PFA) and stored in aliquots at –70°C. For EDIM preparation, the colonic contents of EDIM-infected diarrheic suckling mice were collected and resuspend in PBS followed by centrifugation at 12,000 g for 5 min. The supernatant was filtered through a 0.22 μm filter for sterilization, titrated for median diarrhea-inducing dose (DD50), and stored in aliquots at –70°C.

*Virus titration*

Wa/SA11 strains were titrated by PFA, which was carried out as described (3) with some modification. Briefly, confluent monolayers of MA104 cells in 24-well tissue culture plates were infected with 100 μL supernatants containing infectious RV particles which were serially diluted (10^1^, 10^2^, 10^3^, and 10^4^) with DMEM containing 1 μg/mL trypsin and penicillin (10,000 U/mL)-streptomycin (5 µg/mL) (DMEM-T) for 1 h at 37°C. Then 400 µL agarose overlay (0.8% agarose in 1×DMEM-T) was added and the plates were incubated at 37℃ and 5% CO_2_ for 5 days. The cells in each well were fixed by 1 mL 4% paraformaldehyde (Sangon Biotech, China) overnight (> 12 h) at room temperature. Then, the paraformaldehyde and agarose were removed and the monolayers were stained with 0.5 ml of a 1% (v/v) crystal violet (Sangon Biotech, China) diluted in distilled water for 10 min at room temperature. Then, the plates were drained and dried, and the plaques were enumerated. As the EDIM strain is not able to grow in cell culture, it was titrated by qPCR analysis as described (4) with EDIM VP6 gene as the target. A series of copies (1×10^3^, 1×10^4^, 1×10^5^, 1×10^6^, and 1×10^7^) of EDIM strain were orally inoculated to 7-day-old BALB/c mice and diarrhea was examined daily by gentle palpation of the abdomen till 4 days post infection, and a median diarrhea-inducing dose (DD50) of EDIM (2×10^5^ copies) was calculated as described (5).

*Cytotoxicity assay*

The 50% cytotoxic concentration (CC50) values were determined by nonlinear regression (curve fit) analysis in GraphPad Prism 9.0 (GraphPad Software). Dose-response data (drug concentrations as X-axis and normalized cell viability percentages as Y-axis) were fitted to a Dose-response Inhibition model ("[Inhibitor] vs. normalized response-Variable slope"). The curve was constrained to a bottom limit of 0% viability. The CC50 value was automatically calculated as the X-intercept at Y=50%. Model adequacy was validated using goodness-of-fit metrics (R²≥0.95), 95% confidence intervals, and residual distribution analysis. Final CC50 values were derived from three independent biological replicates and reported as mean ± SEM.

*Flow cytometry analysis*

Differentially-treated MA104 or Caco-2 cells were infected with RV Wa strain (MOI=1). At 20 h p.i. the infected cells were digested with 0.25% trypsin-EDTA, the cells were fixed with 4% paraformaldehyde for 15 min, permeabilized with 1% Triton-X-100 (Sigma-Aldrich, USA) for 7 min, quenched with 50 mM NH_4_Cl for 10 min, and washed for 3 min in PBS twice. Cells were stained with FITC-labeled goat anti-RV polyclonal antibodies (Virostat, USA) (1:100) for 1 h at room temperature, and the frequency of infected cells was analysed with BD Canton II flow cytometry (BD, USA).

*RNA-sequencing (RNA-seq) analysis*

The enriched mRNA was fragmented into short fragments using fragmentation buffer and reversely transcribed into cDNA by using NEBNext Ultra RNA Library Prep Kit for Illumina (NEB, USA). The purified double-stranded cDNA fragments were end repaired, A base added, and ligated to Illumina sequencing adapters. The ligation reaction was purified with the AMPure XP Beads (1.0X), and polymerase chain reaction (PCR) amplified. The resulting cDNA library was sequenced using Illumina Novaseq6000 by Gene Denovo Biotechnology Co. (Guangzhou, China). Raw data (raw reads) of fastq format were further filtered by fastp (version 0.18.0). In this step, clean data (clean reads) were obtained by removing reads containing adapter, reads containing ploy-N and low-quality reads from raw data. Short reads alignment tool Bowtie2 (version 2.2.8) was used for mapping reads to ribosome RNA (rRNA) database. The rRNA mapped reads then will be removed. The remaining clean reads were further used in assembly and gene abundance. Reference genome Homo sapiens Ensembl_release110 and gene model annotation files were downloaded from genome website directly. Index of the reference genome was built using HISAT2.2.4 and paired-end clean reads were aligned to the reference genome using HISAT2.2.4. The mapped reads of each sample were assembled by using StringTie v1.3.1 in a reference-based approach. For each transcription region, a FPKM (fragment per kilobase of transcript per million mapped reads) value was calculated to quantify its expression abundance and variations, using RSEM software. Differential expression analysis was performed using the DESeq2 R package (1.16.1), RNAs differential expression analysis was performed by DESeq2 software between two different groups. The genes/transcripts with the parameter of false discovery rate (FDR) below 0.05 and absolute fold change ≥ 2 was considered differentially expressed genes/transcript.

*qPCR* *analysis*

RV-infected Caco-2 cells or intestines form RV-infected suckling mice were lysed in Trizol reagents for RNA extraction and RNA was further purified using RNeasy Plus Micro or Mini Kit (Qiagen). 0.5 µg of total RNA was reverse transcribed Using HiScript® II Q RT SuperMix for qPCR (+gDNA wiper) Kit (Vazyme, China), and qPCR was performed using ChamQ qPCR SYBR Green Master Mix (Vazyme, China) with corresponding primers on a CFX96 Touch Real-Time System (Bio-Rad). Primers used for amplifications are listed in Supplemental table 1, from which human or mouse β-actin was selected as internal reference. Relative gene expression was calculated via the ΔΔ*Ct* method with normalization to β-actin. Relative gene expression=2^−ΔΔ^*^Ct^*, where ΔΔ*Ct*=(*Ct*_target,sample_​−*Ct*_β-actin,sample_​)−(*Ct*_target,control_​−*Ct*_β-actin,control_).

*ELISA assay*

Viral antigens in the supernatant were detected with enzyme-linked immunosorbent assay (ELISA) kits for RV (CUSABIO, China) following manufacturer's instructions. Briefly, all ELISA reagents were equilibrated to room temperature for ≥30 minutes. Negative/positive controls and samples (n=3) were loaded (50 μL/well) with a blank control, followed by addition of 50 μL enzyme conjugate (excluding the blank). The plates were sealed and incubated at room temperature for 15 minutes. After discarding the liquid, the plates were washed 10 times with deionized water (30 seconds/wash) and dried by blotting. Substrates A and B (50 μL each) were added, mixed gently, and incubated in the dark for 10 minutes. The reaction was stopped with 50 μL of stop solution. Absorbance was measured at 450 nm (reference 650 nm) within 10 minutes, and blank-subtracted OD values were analyzed.

**References**

1. Kaljot KT, Shaw RD, Rubin DH, Greenberg HB. 1988. Infectious rotavirus enters cells by direct cell membrane penetration, not by endocytosis. J Virol 62:1136-44.

2. Rigo-Adrover MDM, Knipping K, Garssen J, Saldana-Ruiz S, Franch A, Castell M, Perez-Cano FJ. 2019. Rotavirus Double Infection Model to Study Preventive Dietary Interventions. Nutrients 11.

3. Willoughby RE, Yolken RH, Schnaar RL. 1990. Rotaviruses specifically bind to the neutral glycosphingolipid asialo-GM1. J Virol 64:4830-5.

4. Zhao W, Xia M, Bridges-Malveo T, Cantu M, McNeal MM, Choi AH, Ward RL, Sestak K. 2005. Evaluation of rotavirus dsRNA load in specimens and body fluids from experimentally infected juvenile macaques by real-time PCR. Virology 341:248-56.

5. Bell LM, Clark HF, O'Brien EA, Kornstein MJ, Plotkin SA, Offit PA. 1987. Gastroenteritis caused by human rotaviruses (serotype three) in a suckling mouse model. Proc Soc Exp Biol Med 184:127-32.

**Supplementary figures**


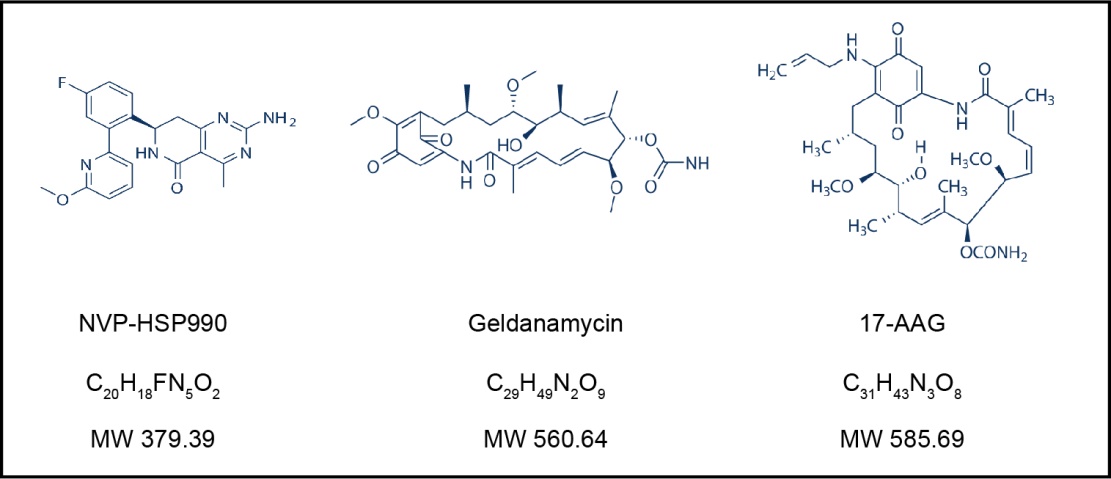


**Supplementary Fig. 1. Chemical structures of NVP-HSP990, Geldanamycin, and 17-AAG.**


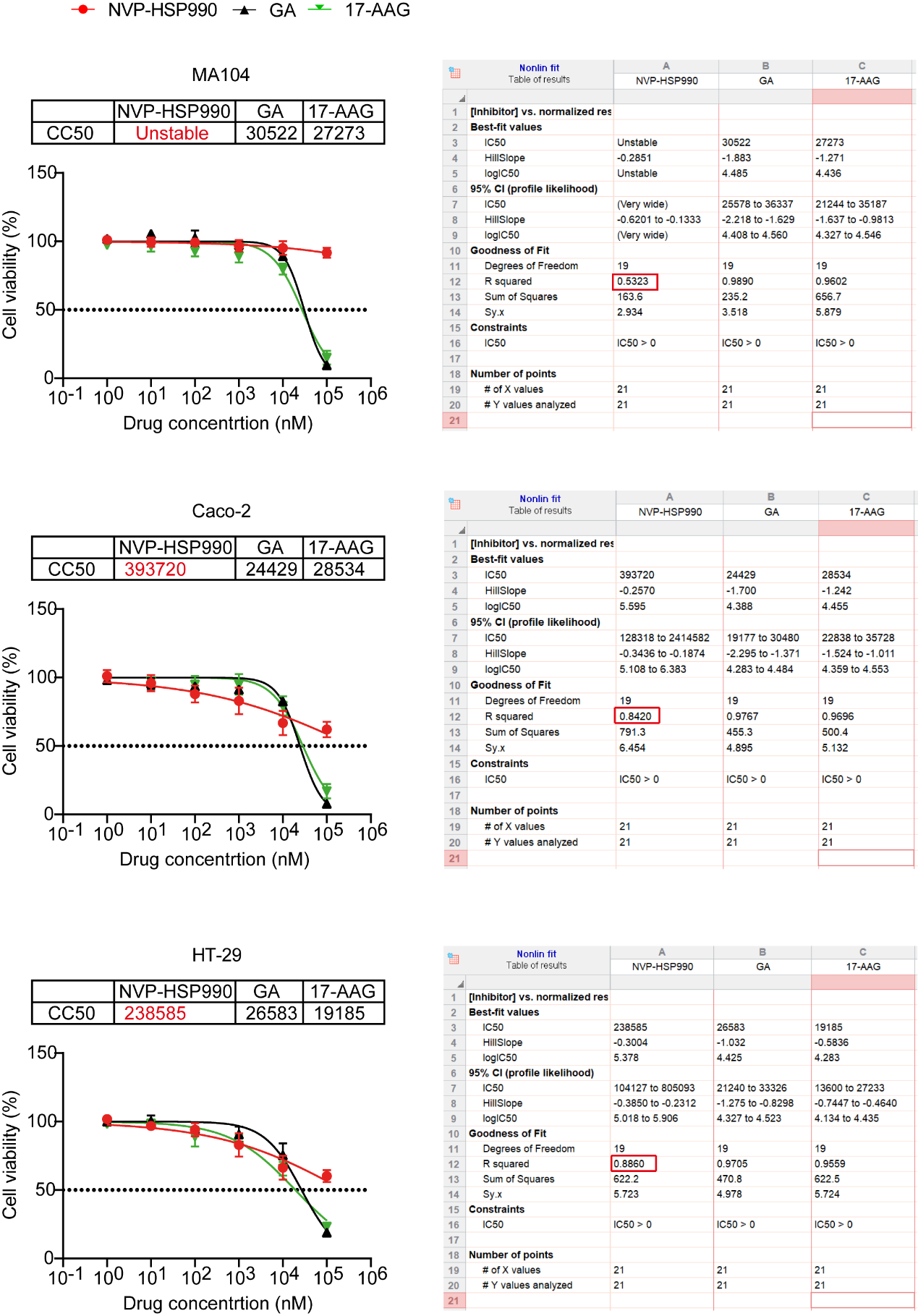


**Supplementary Fig. 2. Cytotoxicity of different HSP90 inhibitors.** (A) Plots (left) and related Nonlin fit analysis (right) for cell viability of MA104, Caco-2 and HT-29 cells after treatment with NVP-HSP990, GA or 17-AAG at indicated concentrations for 24 h. Cell viability was tested using CCK-8 assay and CC50 values are indicated on the top of each plot. Red numbers indicate inaccurate CC50 values, and red boxes highlight R^2^ values that fail to meet the criteria (>0.95). The experiments were performed in triplicate and the data are presented as mean ± SEM and are representative of 4 independent experiments.


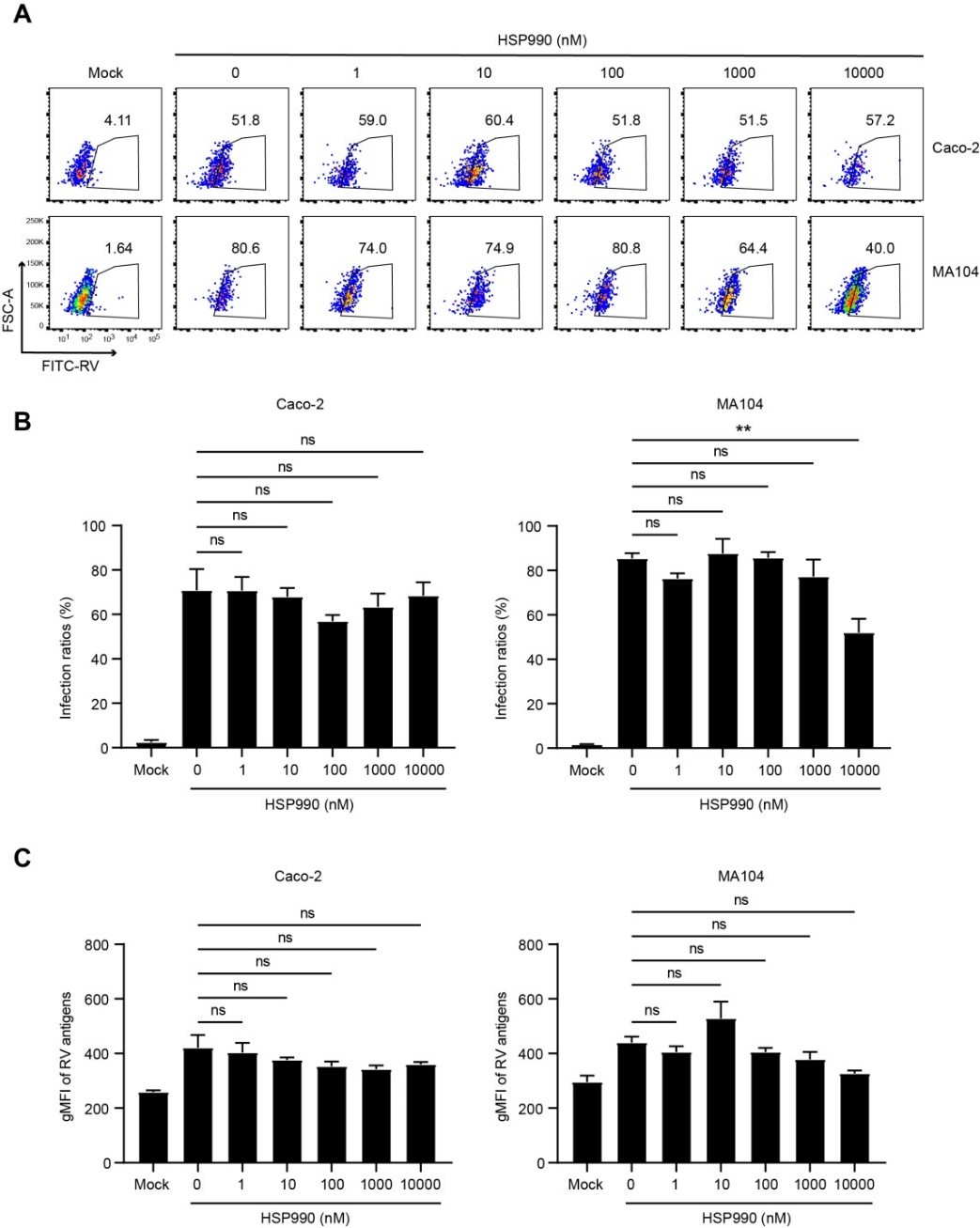


**Supplementary Fig. 3. HSP990 did not influence the establishment of RV infections.** Caco-2 or MA104 cells were treated with a series of NVP-HSP990 concentrations (0, 1, 10, 100, 1000, 10000 nM) for 2 h before RV Wa strain infection (MOI=1) respectively. After washing with 0.01 M PBS for 3 times, the cells were cultured in DMEM for 20 h and then harvested for FACS analysis of infection ratios and viral antigen expression. Representative count plots (A) and statistics of infection ratios (B) and expression of RV antigens (C) are shown. Data are presented as mean ± SEM and are representative of two independent experiments. ns: no significance, ***P* < 0.01 (one-way ANOVA test).


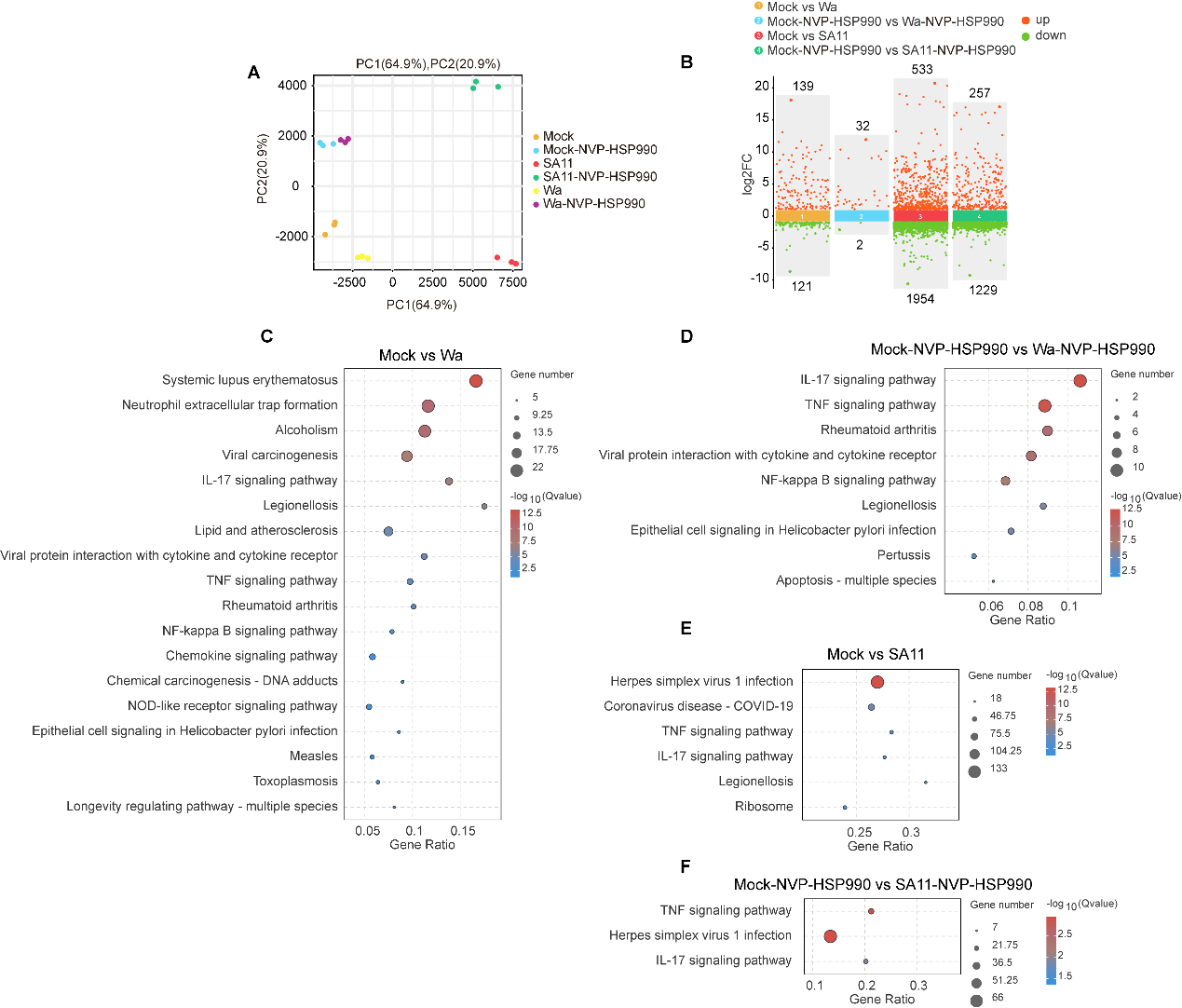


**Supplementary Fig. 4.** **NVP-HSP990 eliminated the effects of RV infection on host cell signaling pathways.** Caco-2 cells were mock infected with PBS or infected with RV Wa or SA11 strains (MOI=3) and further cultivated with DMEM containing 100 nM HSP990 or equal volume of DMSO as control for 24 hours. Then, the infected cells were harvested for RNAseq analysis. PCA analysis (A), multiple differential scatter plots of compared groups (B), and bubble charts of differential KEGG pathways (ranked by descending Q values, Q value < 0.05, Gene ratio > 0.05) in compared groups (C-F) are shown.


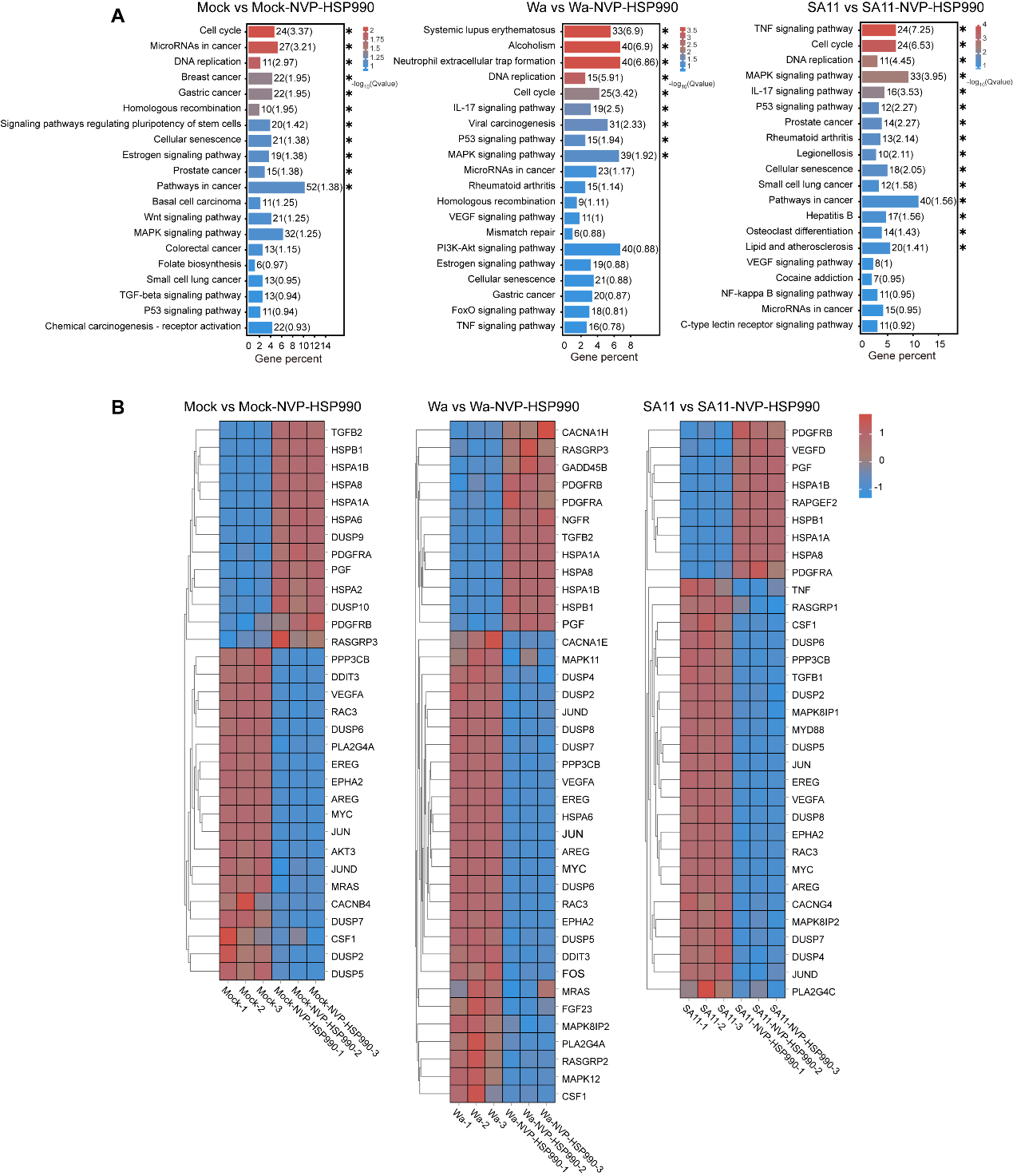


**Supplementary Fig. 5.** **NVP-HSP990 modulated MAPK signaling pathways in Caco-2 cells.** (A) Bar charts of top 20 (ranked by descending Q values) differential KEGG pathways between compared groups. *: Q value < 0.05. (B) Heat maps of differentially expressed genes belonging to MAPK signaling pathway.


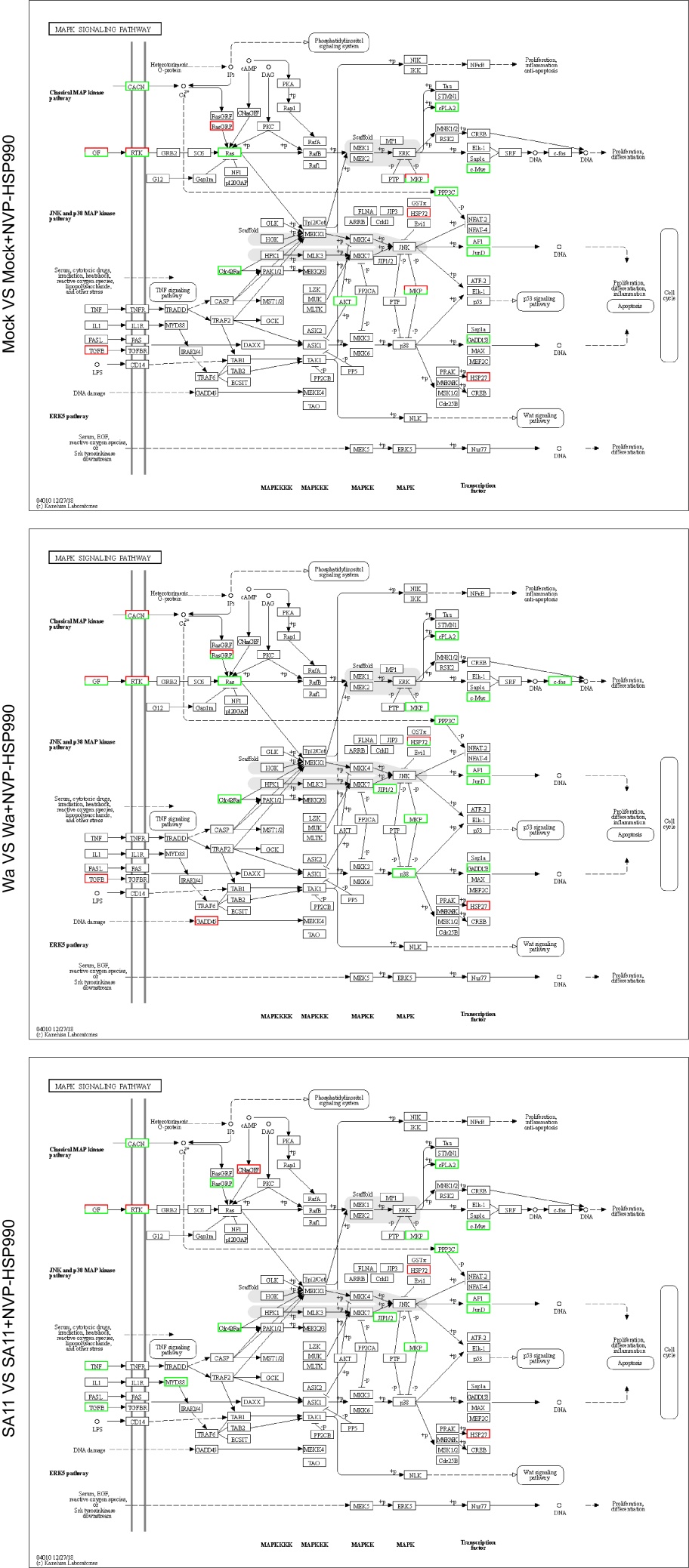


**Supplementary Fig. 6. Altered MAPK signaling pathway (KEGG) in mock- or RV- infected Caco-2 cells by NVP-HSP990.** Red frames indicate up-regulated gene, while green frames indicate down-regulated gene.


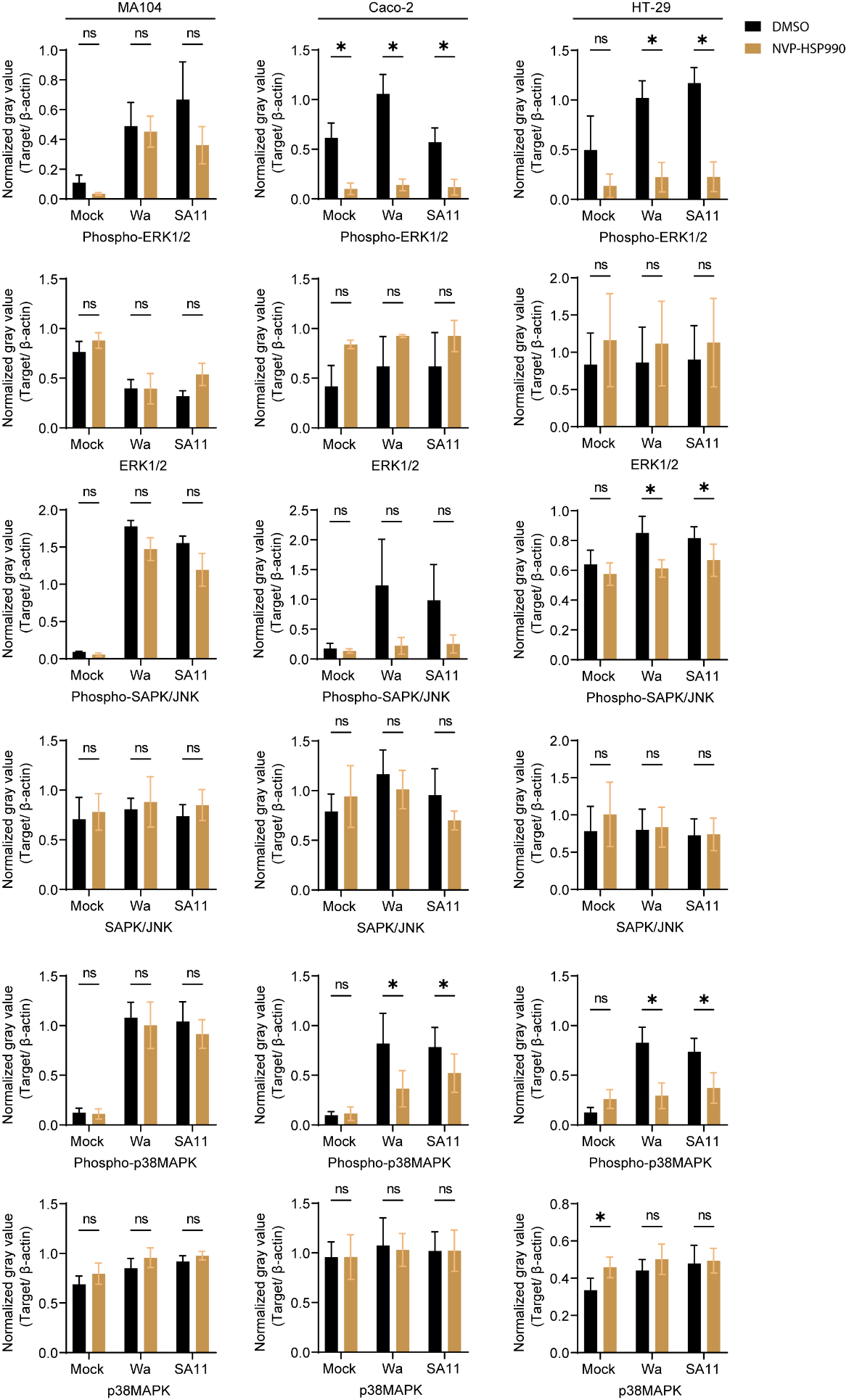


**Supplementary Fig. 7. NVP-HSP990 inhibited MAPK activation.** MA104, Caco-2, and HT-29 cells were mock infected with PBS or infected with RV Wa or SA11 strains (MOI=3), followed by treatment of 100 nM HSP990 (+) or equal volume of DMSO as control (-) for 20 h. Then the infected cells were harvested for WB analysis of MAPK components. The grayscale values of WB bands were quantified, and the relative protein levels of MAPK components were normalized to β-actin. Data are presented as mean ± SEM (n = 3 independent experiments). ns: no significance, **P* < 0.05 (paired Student’s t-test).


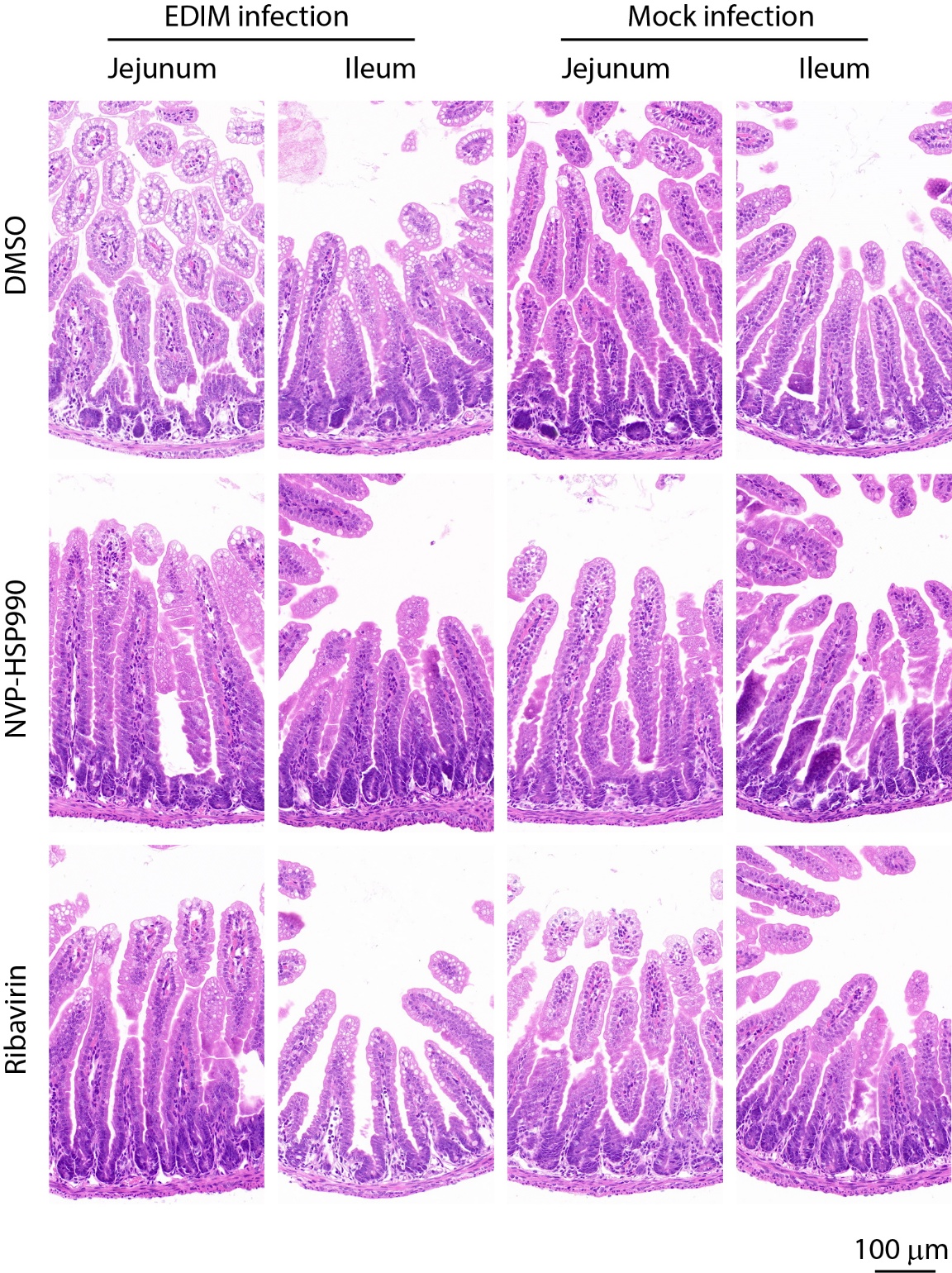


**Supplementary Fig. 8. NVP-HSP990 exhibited therapeutic efficacy to RV infection.** 7-day-old BALB/c suckling mice were orally inoculated with 10×DD50 RV EDIM strain. At 2 d p.i. when diarrhea occurred in all mice, the mice were orally treated with 0.2 mg/kg/day NVP-HSP990, 30 mg/kg/day ribavirin, or equal amount of DMSO as control. After 3 days’ drug treatment, mice intestines (jejunum and ileum) were subjected to histopathological analysis with hematoxylin/eosin staining (n = 3 mice/group). Data are representative of two independent experiments.


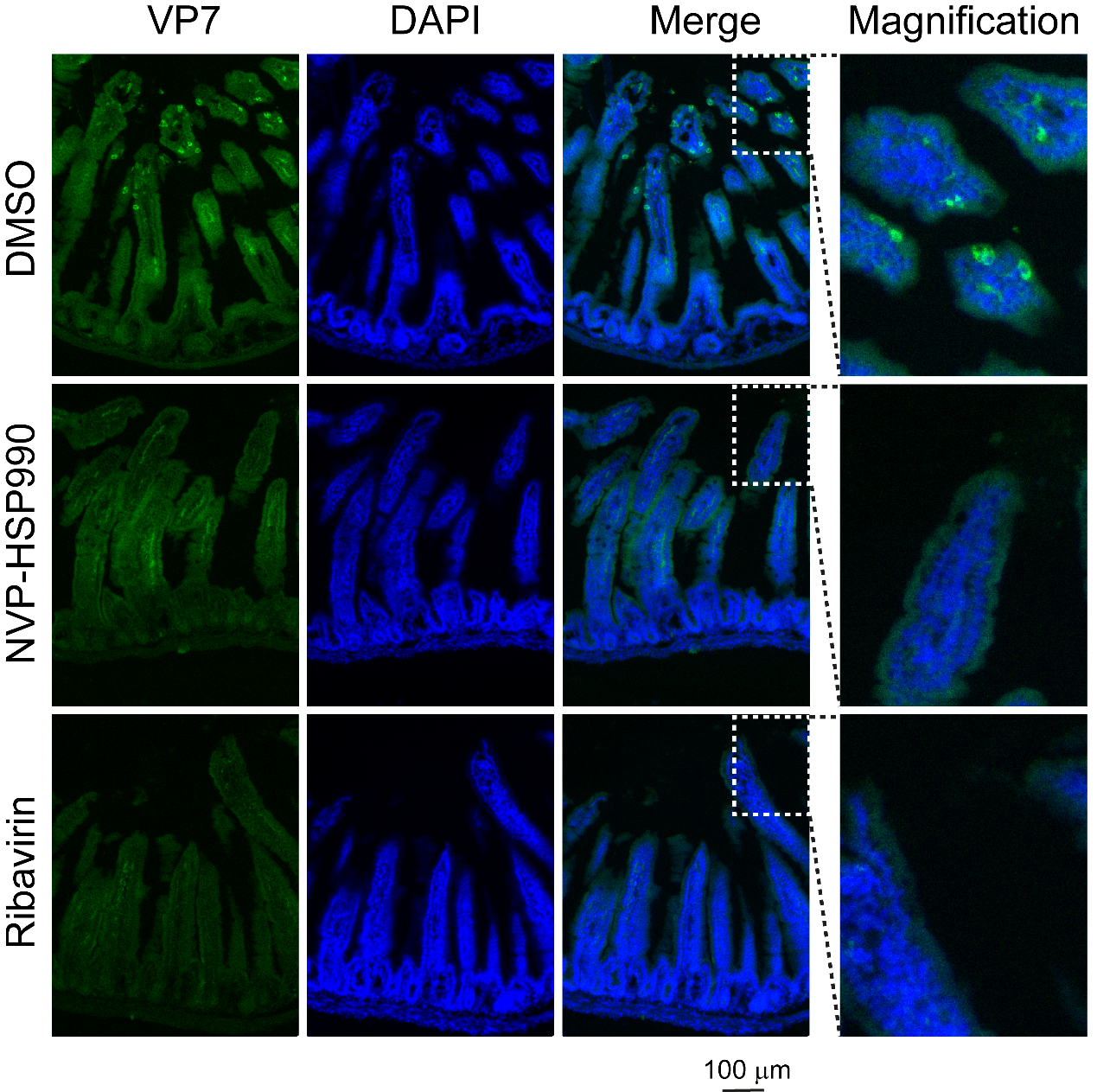


**Supplementary Fig. 9. NVP-HSP990 exhibited therapeutic efficacy to RV infection.** 7-day-old BALB/c suckling mice were orally inoculated with 10×DD50 RV EDIM strain. At 2 d p.i. when diarrhea occurred in all mice, the mice were orally treated with 0.2 mg/kg/day NVP-HSP990, 30 mg/kg/day ribavirin, or equal amount of DMSO as control. After 3 days’ drug treatment, mice intestines (jejunum) were subjected to histopathological analysis of EDIM VP7 (green) and DAPI staining of nucleus (blue) (n = 3 mice/group). Data are representative of two independent experiments.
