# Supplementary material for: A small-molecule HSP90 inhibitor, NVP-HSP990, alleviates rotavirus infection
Source: J Virol. 2025 Dec 10;100(1):e01883-25. doi: 10.1128/jvi.01883-25 (PMC12817916; doi:10.1128/jvi.01883-25)
Supplement: Text S2 — Antibodies and reagents. [file jvi.01883-25-s0002.docx]

Antibodies and Reagents

| ANTIBODIES or REAGENTS | SOURCE | IDENTIFIER |
| --- | --- | --- |
| Antibodies | | |
| Cy3-conjugated goat anti rabbit antibodies | Beyotime | Cat# A0516 |
| FITC-labeled goat anti-RV polyclonal antibodies | Virostat | Cat# 0503 |
| FITC-labeled goat anti-Rabbit antibodies | eBioscience | Cat# 11-4839-81 |
| Horseradish peroxidase (HRP)-conjugated anti-mouse IgG | CST | Cat# 7076S |
| Horseradish peroxidase (HRP)-conjugated anti-rabbit IgG | CST | Cat# 7074S |
| Mouse mAb to b-actin | Servicebio | Cat# GB15001 |
| p38 MAPK (D13E1) XP® Rabbit mAb | CST | Cat# 8690T |
| p44/42 MAPK (Erk1/2) (137F5) Rabbit mAb | CST | Cat# 4695T |
| Phospho-p38 MAPK (Thr180/Tyr182) (D3F9) XP® Rabbit mAb | CST | Cat# 4511T |
| Phospho-p44/42 MAPK (Erk1/2) (Thr202/Tyr204) (D13.14.4E) XP® Rabbit mAb | CST | Cat# 4370T |
| Phospho-SAPK/JNK (Thr183/Tyr185) (81E11) Rabbit mAb | CST | Cat# 4668T |
| Rabbit anti Claudin-1 monoclonal antibody | CST | Cat# 13255 |
| Rabbit anti CD2AP polyclonal antibodies | CST | Cat# 2135 |
| Rabbit anti-RV VP6 polyclonal antibodies | CUSABIO | Cat# CSB-PA542165LA01ROH |
| Rabbit anti-RV VP7 polyclonal antibodies | CUSABIO | Cat# CSB-PA318053LA01ROH |
| Rabbit anti SAPK/JNK polyclonal antibodies | CST | Cat# 9252T |
| Rabbit anti ZO-1 monoclonal antibody | CST | Cat# 13663S |
| Rabbit anti ZO-2 polyclonal antibodies | CST | Cat# 2847 |
| Rabbit anti ZO-3 monoclonal antibody | CST | Cat# 3704 |
| Reagents | | |
| 17-AAG | Selleck | Cat# S1141 |
| 2-(4-Amidinophenyl)-6-indolecarbamidine dihydrochloride (DAPI) | Sigma-Aldrich | Cat# D9542 |
| ChamQ SYBR qPCR Master Mix | Vazyme | Cat# Q311-02/03 |
| BeyoECL Plus | Beyotime | Cat# P0018S |
| Bovine Serum Albumin (BSA) | Beyotime | Cat# ST2254 |
| CCK-8 reagents | Beyotime | Cat# C0038 |
| Crystal violet | Sangon Biotech | Cat# A600331 |
| Dimethyl Sulfoxide (DMSO) | Sigma-Aldrich | Cat# D8418 |
| Dithiothreitol (DTT) | Sangon Biotech | Cat# A620058 |
| DMEM (2X) | Procell | Cat# PM150221 |
| DMEM (high glucose) | Invitrogen | Cat# 11995065 |
| Eosin | Beyotime | Cat# C0109 |
| Fetal bovine serum | Gibco | Cat# A5256701 |
| Geldanamycin (GA) | Selleck | Cat# S2713 |
| Hematoxylin | Beyotime | Cat# C0107 |
| HiScript® II Q RT SuperMix for qPCR (+gDNA wiper) Kit | Vazyme | Cat# R223-01 |
| NEB Next Ultra RNA Library Prep Kit for Illumina | NEB | Cat# 7530 |
| NVP-HSP990 | Selleck | Cat# S7097 |
| Paraformaldehyde | Sangon Biotech | Cat# A500684 |
| Percoll | cytiva | Cat# 17089190 |
| Polyethylene glycol (PEG, MW6,000) | Sangon Biotech | Cat# A610432 |
| Prolong(R) Gold Antifade Reagent | CST | Cat# 9071S |
| Protease inhibitor cocktail | Thermo | Cat# 87786 |
| PVDF membranes | Millipore | Cat# IPVH00010 |
| Ribavirin | Selleck | Cat# S2504 |
| RIPA Lysis Buffer | Beyotime | Cat# P0013B |
| RV antigen ELISA kit | CUSABIO | Cat# CSB-E05033h |
| RV antigen ELISA kit | CUSABIO | Cat# CSB-EQ027718MO |
| Triton-X-100 | Sigma-Aldrich | Cat# T8787 |
| Trizol reagents | Invitrogen | Cat# 12183555 |
| Trypsin | Gibco | Cat# 15090046 |
