# Supplementary material for: A small-molecule HSP90 inhibitor, NVP-HSP990, alleviates rotavirus infection
Source: J Virol. 2025 Dec 10;100(1):e01883-25. doi: 10.1128/jvi.01883-25 (PMC12817916; doi:10.1128/jvi.01883-25)
Supplement: Table S1 — Primers used in qPCR analysis. [file jvi.01883-25-s0003.docx]

Supplemental table 1: Primers used in qPCR analysis.

| Primer name | Forward primer (5’ – 3’) | Reverse primer (5’ – 3’) |
| --- | --- | --- |
| VP2 for Wa/SA11 | ttgcttgcgttcgtttcagc | tggggttggcgtttacagtt |
| VP6 for Wa/SA11 | tgctattaacgcaccagcca | aacctttccgcgtctggtag |
| NSP4 for Wa | acgtcagctggagatgattga | tgcagtcacttctgacggtt |
| NSP4 for SA11 | gtgcaaacgacaggcgaa | catcaggtccgatggtgctta |
| NSP5 for Wa | tgaggatgaaacaagtcgca | cacaaaacgggagtgggga |
| NSP5 for SA11 | gcactacccaagaattgaagca | agctccctagtgtgttccca |
| VP6 for EDIM | acttaccaggccagattcgg | acttgcatccgcgagaact |
| NSP4 for EDIM | tccattctgagagagcgcgt | aacgtgaccaccaccctctt |
| Mouse β-actin | aatcgtgcgtgacatcaaag | ggattccatacccaagaagg |
| Human β-actin | tcaccatggatgatgatatcgc | aatccttctgacccatgcc |
