# Supplementary material for: A small-molecule HSP90 inhibitor, NVP-HSP990, alleviates rotavirus infection
Source: J Virol. 2025 Dec 10;100(1):e01883-25. doi: 10.1128/jvi.01883-25 (PMC12817916; doi:10.1128/jvi.01883-25)
Supplement: Table S2 — CC50, IC50, and SI values. [file jvi.01883-25-s0004.docx]

Supplementary table 2: CC50, IC50 and SI values

| Agents | Cell lines | CC50 (µM) | Viruses | IC50 (nM) | SI | SI 95 % CI |
| --- | --- | --- | --- | --- | --- | --- |
| NVP-HSP990 | MA104 | >100 | Wa | 0.96±0.13 | > 7.30×10⁴ | NA |
|  |  |  | SA11 | 0.84±0.17 | > 7.28×10⁴ | NA |
|  | Caco-2 | >100 | Wa | 4.00±1.28 | > 1.13×10⁴ | NA |
|  |  |  | SA11 | 7.47±4.84 | > 2.66×10³ | NA |
|  | HT-29 | >100 | Wa | 4.69±0.90 | > 1.30×10⁴ | NA |
|  |  |  | SA11 | 3.85±1.16 | > 1.16×10⁴ | NA |
| GA | MA104 | 26.58±5.55 | Wa | 10.46±3.57 | 2.59×10³ | 9.85×10² – 6.80×10³ |
|  |  |  | SA11 | 3.01±0.77 | 8.84×10³ | 4.01×10³ – 1.95×10⁴ |
|  | Caco-2 | 19.96±3.07 | Wa | 91.71±6.95 | 2.16×10² | 1.50×10² – 3.13×10² |
|  |  |  | SA11 | 83.77±20.63 | 2.42×10² | 1.15×10² – 5.07×10² |
|  | HT-29 | 29.20±5.06 | Wa | 16.50±4.41 | 1.80×10³ | 8.28×10² – 3.90×10³ |
|  |  |  | SA11 | 48.16±34.82 | 7.03×10² | 1.30×10² – 3.79×10³ |
| 17-AAG | MA104 | 23.26±6.72 | Wa | 6.81±2.18 | 3.43×10³ | 1.13×10³ – 1.04×10⁴ |
|  |  |  | SA11 | 3.52±0.60 | 6.44×10³ | 2.89×10³ – 1.44×10⁴ |
|  | Caco-2 | 28.58±9.73 | Wa | 161.77±34.25 | 1.72×10² | 6.82×10¹ – 4.33×10² |
|  |  |  | SA11 | 115.92±33.31 | 2.43×10² | 8.43×10¹ – 7.10×10² |
|  | HT-29 | 20.71±6.03 | Wa | 26.42±20.53 | 9.16×10² | 1.42×10² – 5.91×10³ |
|  |  |  | SA11 | 54.04±7.14 | 3.75×10² | 1.92×10² – 7.31×10² |

CC50 and IC50 are expressed as mean ± SD from 4 and 3 independent experiments, respectively (unpaired design). SI (selectivity index) = geometric mean CC50 / geometric mean IC50; its 95 % CI was estimated by the delta method on log-transformed unpaired data. For agents where CC50 exceeded the maximum tested concentration (100 µM), SI is reported as a lower-bound estimate. NA, not applicable.
